# Roads, Soil, Snow, and Topography Influence Genetic Connectivity: A Machine Learning Approach for a Peripheral American Badger Population

**DOI:** 10.1002/ece3.73467

**Published:** 2026-04-16

**Authors:** Eric C. Palm, Erin L. Landguth, Karina Lamy, Jamieson C. Gorrell, Richard D. Weir, Emma L. Richardson, Krystyn J. Forbes, Helen Davis, Joanna M. Burgar

**Affiliations:** ^1^ School of Public and Community Health Sciences University of Montana Missoula Montana USA; ^2^ Department of Fisheries and Wildlife Michigan State University East Lansing MI USA; ^3^ Ministry of Water, Land, and Resource Stewardship Victoria British Columbia Canada; ^4^ Biology Department Vancouver Island University Nanaimo British Columbia Canada; ^5^ Department of Ecosystem Science and Management University of Northern British Columbia Prince George British Columbia Canada; ^6^ Artemis Wildlife Consultants Duncan British Columbia Canada; ^7^ Thompson Rivers University Kamloops British Columbia Canada

**Keywords:** corridor analysis, landscape genetics, machine learning, peripheral population, spatial cross validation, *Taxidea taxus jeffersonii*

## Abstract

Effective management and conservation of peripheral populations require an understanding of the landscape conditions inhibiting dispersal and spatially explicit predictions of connectivity. Here, we modeled landscape resistance and genetic connectivity for the western population of an American badger subspecies ( *Taxidea taxus jeffersonii* ) across ~170,000 km^2^ in southern British Columbia, Canada, using 116 genetic samples genotyped at 14 microsatellite loci. We used gradient boosting machine models in a corridor‐based approach to predict genetic distances between pairs of individual badgers as a function of landscape variable data. Spatial genetic autocorrelation tests and our top model predicted that genetic similarities of *T. t. jeffersonii* were present up to ~110 km. Gene diversity was lowest in the Cariboo region in the northwest portion of the study area and highest in the Okanagan region in the southeast. Our analyses suggest that the genetic connectivity of *T. t. jeffersonii* was impeded by colluvial soil parent material, geographic distance, steep slopes, and major roads, but was facilitated by organic and fluvial soil parent materials, and areas with relatively little snow cover during winter. Our predictive maps of landscape resistance and connectivity can help guide management actions such as habitat protection and underpass placement on major roads to promote genetic connectivity.

## Graphical abstract

We used a machine‐learning, corridor‐based approach to model landscape resistance and genetic connectivity for an endangered population of American badgers in southern British Columbia, Canada. We found that genetic connectivity in badgers was impeded by colluvial soil parent material, geographic distance, steep slopes, and major roads, but was facilitated by organic and fluvial soil parent materials and areas with relatively little snow cover during winter.

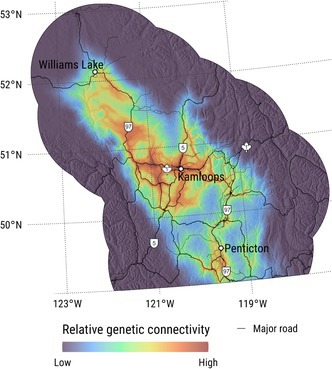

## 1 Introduction

Peripheral populations are geographically separated from and often experience different biotic or abiotic conditions than central populations (Lesica and Allendorf 1995). Conserving peripheral populations can help preserve genetic diversity, increase resilience to environmental change, and maximize the evolutionary potential of a species (Allendorf et al. 2012). However, these populations are often at higher risk of extirpation than central populations due to factors such as geographic and genetic isolation, marginal quality or limited availability of habitat, and genetic drift (Lesica and Allendorf 1995). Human land use may exacerbate threats to peripheral population viability by further degrading and fragmenting habitat and introducing impediments to gene flow (Eckert et al. 2008; Ethier et al. 2012).

Understanding the degree to which landscape attributes, including human land use, influence gene flow in peripheral populations is essential for their successful conservation (Cross et al. 2023). Conservation strategies increasingly incorporate genetic data to help identify potential barriers to movement and use spatially explicit predictions of genetic connectivity to help identify important dispersal corridors (Dickson et al. 2019). Together, this information informs targeted actions to facilitate gene flow and promote long‐term population viability (Vogt et al. 2009).

The westernmost subspecies of American badger ( *Taxidea taxus jeffersonii* ) in southern British Columbia (BC), Canada, includes two endangered peripheral populations whose long‐term viability relies on gene flow and connectivity both within their range and to neighboring populations (Government of Canada 2002; Environment and Climate Change Canada 2023). These two populations consist of an estimated 150–245 badgers in the west and 100–160 badgers in the east, separated by the Selkirk and Monashee Mountains, which represent a major barrier to gene flow (Kyle et al. 2004; Ethier et al. 2012; Environment and Climate Change Canada 2023). Within BC, the western population of *T. t. jeffersonii* is more isolated genetically and geographically than the eastern population, which is likely more closely related to populations in Idaho and Montana (Kyle et al. 2004; Ethier et al. 2012; Ford et al. 2019). Gene flow within the western population is low and asymmetric, generally moving from BC toward the more central, larger populations to the south in Washington (Ford et al. 2019). The combination of low population size and low incoming gene flow in the western population of *T. t. jeffersonii* highlights the need to maintain and restore connectivity throughout its range in BC.

Conservation of *T. t. jeffersonii* in BC is further complicated by the high degree of spatial overlap between badger habitat and human development. Friable soils that badgers require for burrowing include those with fluvial, glacial fluvial, and lacustrine parent materials, which are primarily confined to valley bottoms (Apps et al. 2002; Hoodicoff 2003; Weir et al. 2003, 2017; Kinley and Newhouse 2008; Klafki 2014), where human settlements and extensive road networks pose major risks to their survival and may limit their connectivity. Roads are the largest source of direct mortality for *T. t. jeffersonii* (Environment and Climate Change Canada 2023). Continued human population growth projected for the region (BC Stats 2024) and concomitant increases in vehicle traffic may increase badger mortality risk and further impede connectivity.

Modeling landscape resistance in southern BC would clarify the influence of roads on badger gene flow and inform conservation efforts to minimize their negative effects on connectivity, satisfying a priority objective of the federal Recovery Strategy for *T. t. jeffersonii* (Environment and Climate Change Canada 2023). Currently, the Recovery Strategy identifies and maps critical habitat that is necessary for their dispersal and reproduction (“safe movement” critical habitat) by using a combination of data from habitat association studies, surveys, and public sightings (Environment and Climate Change Canada 2023). Although these data can serve as proxies for functional connectivity, dispersing individuals may select habitat differently when moving within their home range, especially in highly mobile species such as badgers (Abrahms et al. 2017). Genetic data provide an index of successful dispersal and can help clarify the relative contributions of movement capability versus habitat preferences in determining species' spatial genetic structure and functional connectivity (Kierepka and Latch 2016a).

In this study, we mapped genetic connectivity, the degree to which gene flow affects evolutionary processes within subpopulations (Lowe and Allendorf 2010), for the western population of *T. t. jeffersonii* in southern BC. We used a corridor‐based approach to model genetic distances between pairs of individual badgers as a function of landscape variable data. We used these models to objectively parameterize landscape resistance surfaces, from which we predicted genetic connectivity. Based on existing studies of *T. t. jeffersonii* habitat selection in the region, we hypothesized that human development, topography, soil parent material, land cover, and canopy cover were important predictors of landscape resistance and genetic connectivity. We predicted that major roads, less friable soils (such as colluvial parent materials along hillsides and mountainous areas), steep slopes, and dense forests would impede dispersal and gene flow. For peripheral populations, maps of genetic connectivity can help prioritize areas for targeted conservation actions that facilitate genetic exchange, thereby improving the ability of those populations to respond to future environmental perturbations and increasing their likelihood of persistence.

## 2 Materials and Methods

### 2.1 Study Area

**FIGURE 1:**
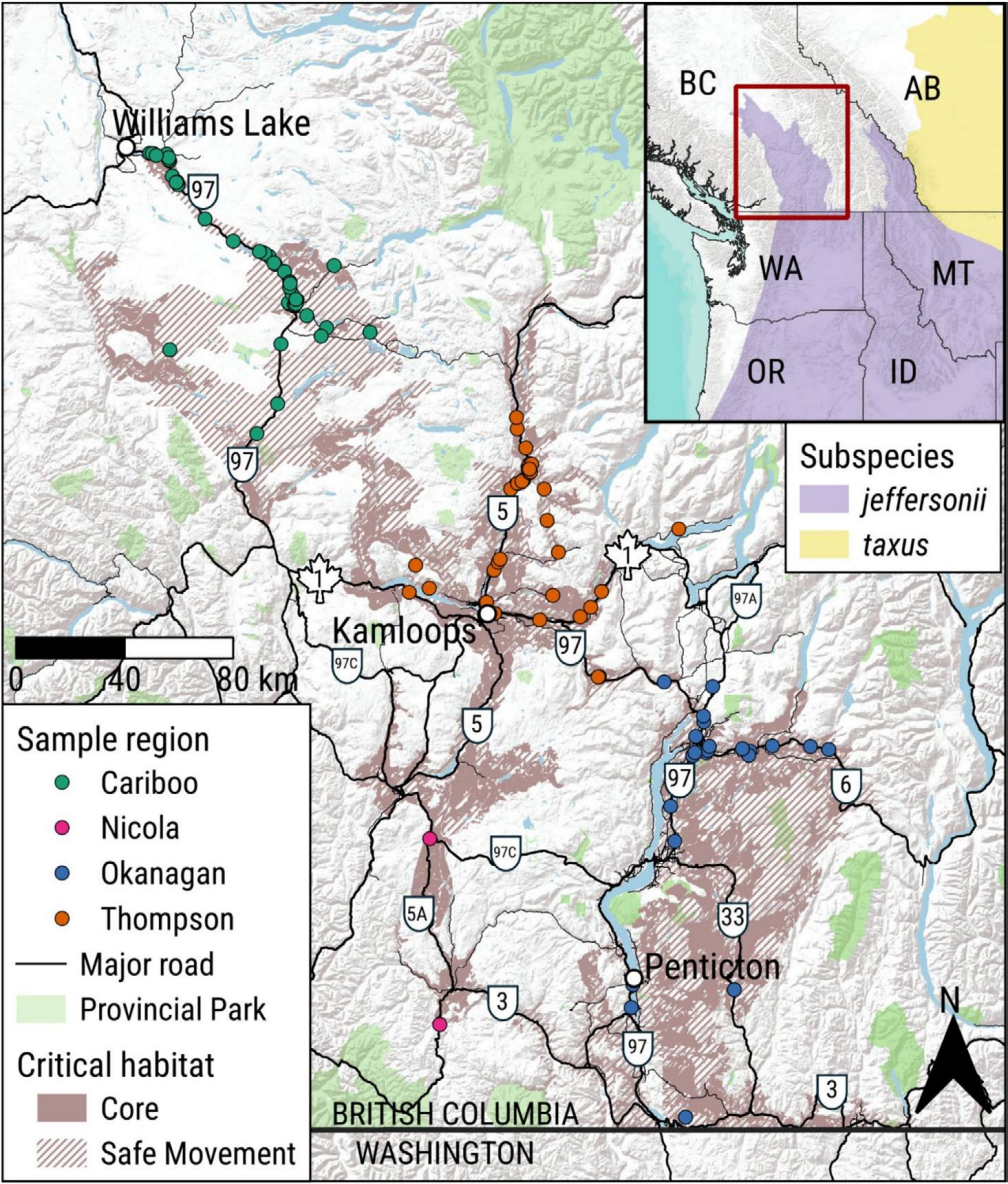
Locations of 116 American badger ( *Taxidea taxus jeffersonii* ) genetic samples (shown by region) collected between 2008 and 2023 in southern British Columbia, Canada, used in landscape genetics models, along with federally identified critical habitat for the western population of *T. t. jeffersonii* in Canada. Selected major highways are labeled by route number. Inset depicts distribution of badger subspecies in the northwest USA and southwest Canada.

Our study area included approximately 170,000 km^2^ of southern BC from Williams Lake to Osoyoos, an area encompassing the entire range of the western population of *T. t. jeffersonii* in BC with a 110‐km buffer (Figure 1). We divided the area into four regions (Figure 1), which corresponded to spatially distinct, at‐risk ecological communities used in the federal Recovery Strategy for *T. t. jeffersonii*: Cariboo, Thompson, Nicola, and Okanagan (Environment and Climate Change Canada 2023; Government of British Columbia 2025). Major highways (with maximum annual average daily traffic volumes from 2010 to 2015; British Columbia Ministry of Transportation and Infrastructure 2022) by region include TransCanada Highway 1 in the Thompson (east–west; 23,000), Highways 5 and 5A (north–south) in the Thompson (29,000) and Nicola (1200) regions, and Highways 97, 97A, and 97C (north–south) through the Cariboo (7000), Thompson (23,000), and Okanagan (56,000) regions. The Cariboo region in the northwest has colder, snowier winters, is more heavily forested, and has a higher density of wetlands than the more southern Thompson, Nicola, and Okanagan regions (Packham and Hoodicoff 2004; Symes 2013). The principal biogeoclimatic ecosystem classification zones within the study area are Interior Douglas‐fir, Bunchgrass, and Montane Spruce (MacKenzie and Meidinger 2018). Only a small portion of the area inhabited by the western population of *T. t. jeffersonii* falls within protected areas, including ~7% of grasslands in the Okanagan region (Grasslands Conservation Council of British Columbia 2004; Klafki 2014).

### 2.2 Genetic Sample Collection and Analyses

We collected genetic samples from 116 badgers from 2008 to 2023 across all four regions (Cariboo: *n* = 48; Thompson: *n* = 40; Okanagan: *n* = 26; Nicola: *n* = 2). Samples were collected opportunistically, primarily from road mortalities or other causes of mortality, hair snagging surveys, and live captures. Most samples were collected along major roads in three general areas: (1) Highway 97 in the Cariboo region, (2) Highways 5 and 1 in the Thompson region, and (3) Highways 97 and 6 in the Okanagan region. Of the 77 samples where location precision was recorded, 47 (61%) were estimated to be < 100 m from the collection location, 23 (30%) were < 1 km, and seven (19%) were < 10 km. We note that 64 genetic samples were also used in Ford et al. (2019) (Table S1).

DNA was extracted by Vancouver Island University and Wildlife Genetics International using DNeasy Blood & Tissue kits (Qiagen) following the manufacturer's protocol (Table S1). We used fluorescently labeled forward primers to amplify 14 microsatellite loci, including Tt‐1, Tt‐2, Tt‐3, Tt‐4, and Ma‐1 (Davis and Strobeck 1998), Mvis072 (Fleming et al. 1999), Gg234 (Duffy et al. 1998), Gg443 and Gg465 (Walker et al. 2001), and Tt13, Tt15, Tt20, Tt21, and Tt27 (Rico et al. 2014; Table S2). Reverse primers were pigtailed following the approach recommended by Brownstein et al. (1996) to reduce adenylation. PCR reactions contained 5 μL of Type‐it Microsatellite Multiplex Mastermix (Qiagen) or 5 μL HotStart Taq 2X Mastermix (New England Biolabs), 1 μM of each primer, 2 μL of genomic DNA (approximately 10–80 ng), and ribonuclease‐free water to a final volume of 10 μL. Locus Gg234 had a final concentration of 2.5 mM MgCl_2_ and 1.6 μg/μl bovine serum albumin. Cycling conditions were 95°C for 4 min followed by 35–45 cycles of 95°C for 30 s, a locus‐specific annealing temperature for 30 s, and 68°C for 30 s, with a final extension at 68°C for 1 min (see Table S2 for details on loci). Amplified fragments were analyzed on an ABI 3730 sequencer and manually scored according to length polymorphism using Geneious 10.2 (Kearse et al. 2012). We calculated the number of alleles, observed heterozygosity, unbiased expected heterozygosity, and Wright's fixation index for each locus in GenAlEx v6.5. We also tested for deviations from Hardy–Weinberg equilibrium using exact tests with 1000 Monte Carlo permutations across all loci in the “pegas” R package (Paradis 2010), and for deviations from linkage disequilibrium (or non‐independence of alleles across loci) using the standardized index of association in the “poppr” R package (Kamvar et al. 2014).

### 2.3 Individual‐Based Genetic Differentiation and Spatial Genetic Structure

We used genotyped samples to calculate three genetic distance metrics between all pairs of individual badgers in our dataset with the “gstudio” R package (Dyer 2012). We calculated the proportion of shared alleles (equivalent to 1 − Bray‐Curtis distance; Bray and Curtis 1957; Bowcock et al. 1994), the Queller and Goodnight relatedness estimator (Queller and Goodnight 1989), and Euclidean genetic distance (Excoffier et al. 1992), each of which quantifies genetic dissimilarity (or relatedness) between pairs of individuals with single values and does not assume Hardy–Weinberg equilibrium (Shirk et al. 2017). We ran separate landscape resistance models using each metric as the response variable and compared model performance across metrics (see details below in “Landscape resistance modeling”) for use in our final model. We used Mantel tests for Pearson's correlation and spatial correlograms (“ecodist” package in R; Goslee and Urban 2007) to assess isolation by distance throughout our study area (Legendre and Fortin 1989).

Estimating and visualizing spatial patterns of genetic diversity for imperiled populations can provide increased spatial resolution and fidelity for guiding management and recovery efforts beyond diversity indices summarized within imposed (often artificially or subjectively) discrete boundaries (Shirk and Cushman 2011). We estimated rarefied allelic richness by grouping individuals into overlapping, biologically relevant spatial genetic neighborhoods, an approach that captures spatially complex patterns of gene diversity in clinal or landscape‐driven populations (Shirk and Cushman 2011). We inferred a circular spatial genetic neighborhood size with a radius corresponding to the distance where spatial autocorrelation in genetic distances switched from positive to negative (Waser and Elliott 1991; Shirk and Cushman 2011), and then calculated average rarified allelic richness across loci (El Mousadik and Petit 1996) within these spatial genetic neighborhoods using the ‘hierfstat’ R package (Goudet and Jombart 2021). We required > 10 samples within each spatial genetic neighborhood to calculate rarefied allelic richness.

We also assessed patterns of genetic structure using the clustering software STRUCTURE 2.3.4 (Pritchard et al. 2000). We tested for the presence of one to five clusters (*K*) with 10 iterations of each *K* and used 500,000 Markov Chain Monte Carlo replicates after a burn‐in of 100,000 replicates. We determined the most likely number of clusters by visualizing outputs in StructureSelector (Li and Liu 2018), including Puechmaille (Puechmaille 2016) estimators and methods from Evanno et al. (2005). We generated admixture plots using Clump Ak (Kopelman et al. 2015) and mapped final admixture values. Each cluster was reanalyzed separately using the same parameters to investigate hidden substructure as recommended by Janes et al. (2017).

### 2.4 Landscape Resistance Modeling

We aimed to understand the degree to which landscape factors affected genetic connectivity by modeling genetic distance as a function of geographic distance and landscape features, following the methods of Palm et al. (2023). Briefly, this approach used landscape data summarized along either straight lines or least‐cost paths (LCPs) between pairs of genetic sample locations to model genetic distances and generate spatial predictions of landscape resistance. Past studies using similar analytical approaches found that landscape data summarized along least cost paths better predicted pairwise genetic distances than data summarized along straight lines (Van Strien et al. 2012; Bouyer et al. 2015; Pless et al. 2021; Bishop et al. 2021). Corridor‐based (alternatively, “transect‐based”) approaches with machine learning have been shown to effectively estimate the expected contribution of individual landscape variables to the final multivariate resistance surface and can incorporate spatial cross validation to improve model transferability (Palm et al. 2023; Vanhove and Launey 2023).

We used 13 landscape variables in our models that we hypothesized would impede or promote genetic connectivity for *T.t*. *jeffersonii* in this area based on previous studies of habitat relationships and landscape genetics (e.g., Apps et al. 2002; Hoodicoff 2003; Weir et al. 2003; Klafki 2014; Ford 2017; see Table 1 for details on variables and associated predictions). First, we created straight lines between all pairs of genetic sample locations, buffered each line by 500 m to create a 1000‐m‐wide polygon (i.e., transect), and then calculated the mean value of 180‐m raster pixels for each landscape variable within these straight‐line transects using the “exactextractr” R package (Baston 2021). For binary and categorical variables (e.g., major roads and soil parent materials), the mean value extracted along a transect represented the proportion of the transect covered by that landscape attribute. We used the mean extracted landscape values, along with the geographic distance calculated between pairs of sample locations, as explanatory variables in a regression with pairwise genetic distance as the response variable. Including geographic distance as an explanatory variable helped control for the effect of isolation‐by‐distance and prevent selection of false‐positive landscape variables (Row et al. 2017). This first model (straight‐line model) was necessary to generate an initial spatial prediction of landscape resistance, which represented the predicted degree to which each map pixel inhibited gene flow.

**TABLE 1:** Landscape variables that were hypothesized to influence genetic differentiation and genetic connectivity for American Badgers ( *Taxidea taxus jeffersonii* ) in southern British Columbia, Canada.

Variable(s)	Data type	Landscape resistance prediction	Prediction rationale	Literature cited	Data source
Pairwise geographic distance	Continuous	Greater distance inhibits gene flow	Isolation by distance	Wright (1943)	
Colluvial parent material	Binary	Inhibits gene flow	Rocky material inhibits burrowing	Apps et al. (2002), Weir et al. (2003)	Bulmer et al. (2016)
Fluvial, glaciofluvial, and lacustrine parent materials	Binary	Facilitates gene flow	Friable soil is important for denning and foraging	Weir et al. (2003), Packham and Hoodicoff (2004), Weir and Almuedo (2010), Kinley et al. (2013)	Bulmer et al. (2016)
Organic parent material	Binary	Facilitates gene flow	Commonly den near wetlands in Cariboo region	Packham and Hoodicoff (2004), Klafki (2014)	Bulmer et al. (2016)
Large rivers and water bodies	Binary	Inhibits gene flow	Badgers avoid swimming long distances	Kierepka and Latch (2016a)	Bulmer et al. (2016)
Major roads (primary/secondary highways, freeways)	Binary	Inhibit gene flow	Biggest source of badger mortality; previously shown to inhibit gene flow	Kinley and Newhouse (2009), Klafki (2014), Ford (2017)	Government of British Columbia (2017)
Grassland, shrubland, wetland	Binary	Facilitate gene flow	Badgers select areas within grasslands/open shrubs (and wetlands in the Cariboo) for home ranges	Packham and Hoodicoff (2004), Kinley et al. (2013)	Latifovic et al. (2012)
Slope	Continuous	Steeper slopes inhibit gene flow	Traversing steep slopes is energetically costly	Apps et al. (2002), Kinley et al. (2013)	Derived from Farr et al. (2007)
Canopy cover	Continuous	Higher cover inhibits gene flow	Avoid dense forest with low prey availability	Apps et al. (2002), Hoodicoff (2003), Weir et al. (2003), Kinley and Newhouse (2008), Klafki (2014)	Townshend (2016)
Annual snowfall	Continuous	Snowier areas inhibit gene flow	Snowy areas are often mountainous with steep slopes and closed canopies; deep snow may impede movement	Karasov (1992)	Wang et al. (2016)

Next, we used the predicted resistance surface from the straight‐line model to identify LCPs between the same genetic sample location pairs using the “gdistance” R package (van Etten 2017), buffered the paths by 500 m, and calculated the mean values of landscape variables extracted within these transects. Although LCPs rely on the assumption that animals have complete knowledge of the landscape and choose the path of minimum cumulative resistance, they should be more plausible dispersal routes than straight‐line paths (Adriaensen et al. 2003). We then modeled pairwise genetic distances as a function of the landscape variables summarized along the LCP transect and pairwise geographic distance (LCP model) and generated an updated landscape resistance surface by predicting from this fitted model. We repeated the steps of calculating LCPs using the previous model's predicted resistance surface, summarizing landscape variable values along these buffered paths, and modeling variation in genetic distances until model performance no longer improved (see below for details on performance metric). We fitted separate straight‐line landscape resistance models for each genetic distance metric, including a model for each metric with geographic distance as the only explanatory variable, which provided a basis of comparison for models that included landscape variables.

We modeled genetic distances using gradient boosting machines fitted with the “gbm” R package (GBM; Greenwell et al. 2020). These models can identify complex non‐linear relationships, accommodate multiple collinear explanatory variables, eliminate the need for explanatory variable transformations, fit complex interactions, and make accurate predictions (Friedman 2001; De'ath 2007). We specified GBMs to consider up to two‐way interactions between all variables in the model and incorporated a leave‐one‐cluster‐out spatial cross‐validation (Palm et al. 2023) to ensure that modeled relationships between genetic distance and landscape attributes were not due to spatial clustering of genetic samples or uneven sample sizes across these spatial clusters. We conducted a grid search with a range of possible values for each of the shrinkage rate, number of trees, minimum observations in node, and interaction depth to find the optimal combination of hyperparameters in the GBM models (Bergstra and Bengio 2012). We present the relationships between landscape variables and genetic distance, including statistical uncertainty, using accumulated local effects plots implemented in the ‘ale’ R package (Okoli 2023, 2026).

All GBM models incorporated spatial cross‐validation and variable selection to remove uninformative variables and minimize overfitting (Meyer et al. 2019), helping ensure that relationships between landscape covariates and genetic distance were not limited to localized areas or due to uneven sampling intensity throughout the study area. We assigned each genetic sample to a spatial cluster, and for each cluster, the cross‐validation procedure in the “caret” R package (Kuhn 2008) trained a GBM model using data from all pairwise transects except those where either of the two sample locations belonged to that spatial cluster. We used nearest neighbor distance matching to assign each genetic sample location to a spatial cluster (Milà et al. 2022) with the “knndm” function in the “CAST” R package. This approach ensured that in the cross‐validation process, the training data provided adequate coverage of the study area and that the withheld test data were representative of the prediction locations. We limited our analysis to six spatial clusters, as more clusters resulted in at least one cluster with only two genetic sample locations. Our six spatial clusters contained between 9 and 35 sample locations (Figure S1). The variable selection component of the cross‐validation procedure (implemented using the ‘ffs’ function in the CAST package) trained candidate models with all possible combinations of variables and selected the model with the lowest root mean squared error (RMSE_test_) averaged across all six withheld spatial clusters. Lower average RMSE_test_ values indicated an improved capacity to predict genetic distances of withheld test data and a more generalizable model (Meyer and Pebesma 2021). We selected the model with the lowest RMSE_test_ as the top model. We compared predicted response curves from our model to one with six randomly generated (aspatial) cross‐validation folds to help assess the degree to which our model prevented overfitting. Finally, we ran separate straight‐line models (without feature selection) for the northwest (Cariboo region) and southeast portions (Nicola, Okanagan, and Thompson regions) of our study area to help understand the degree to which relationships between landscape features and genetic distance reflected patterns in one or both areas.

We predicted landscape resistance from each GBM model (straight‐line model and all LCP model iterations) by applying the “predict” function in the “caret” R package to the multiband raster of landscape variables retained in that model. We used a geographic distance raster with the median geographic distance between all pairs of locations. We calculated the relative influence of each variable included in the top‐performing model on the variation in genetic distance using Friedman's (2001) equation for boosted estimates, where influence values are scaled between 0 (least influential) and 100 (most influential). Following recommendations in Beninde et al. (2024), we assessed the convergence of our results across the three genetic distance metrics by calculating pairwise Pearson's correlations between their final landscape resistance rasters. Finally, we fitted separate straight‐line GBM models (with spatial cross validation and variable selection) using each of the three genetic distance metrics as the response variable and directly compared their performance. Because these metrics are measured on different scales, we calculated an “adjusted” average RMSE_test_, where for each metric, we normalized genetic distances to be between 0 and 1 prior to modeling and then divided the average RMSE_test_ by that average normalized genetic distance.

### 2.5 Predicting Genetic Connectivity

We mapped predicted genetic connectivity by applying resistant kernels (Compton et al. 2007) to depict corridors and movement areas predicted from the top‐performing landscape resistance model. Resistant kernels depict corridors and movement areas by estimating the relative probability of individuals dispersing from focal cells to other points on the landscape. The algorithm created an individual kernel from each of 2000 random locations within the distribution of the western population of *T. t. jeffersonii* by using the landscape resistance raster pixel values to calculate the LCP to every other cell in the landscape within a specified cost‐distance threshold. Individual kernels were then summed at each raster pixel. Wider kernel spread occurred in areas of lower landscape resistance, translating into higher predicted values of genetic connectivity. We normalized the input resistance surface to be between 1 and 100 to simplify choosing a cost‐distance threshold (see below), set all water bodies > 3 km^2^ to have a resistance of 100 before applying resistant kernels in UNICOR (Landguth et al. 2012). We used a linear transform function and a threshold of 1,100,000 cost‐distance units at a low predicted resistance value of 10, which prevented modeled dispersal distances from exceeding badgers' maximum recorded dispersal distance of ~110 km (Messick and Hornocker 1981).

## 3 Results

### 3.1 Genetic Summary Statistics and Spatial Genetic Structure

We observed considerable genetic variation among the samples that we collected from throughout the western population. We observed between 4 and 11 alleles across the 14 microsatellite loci, and the proportion of missing genotypes ranged from 0 to 0.03 (pooled across loci = 0.01). Observed and expected heterozygosity ranged from 0.609 to 0.853 and from 0.614 to 0.858, respectively (Table S2). Eight of the 14 loci did not stray significantly from the null expectation of Hardy–Weinberg equilibrium (Table S3 and Figure S2). The deficit of heterozygotes in the remaining loci may reflect inbreeding or the presence of null alleles. Estimated frequencies of null alleles ranged from 0 to 0.103 (mean = 0.028 ± 0.033) across the 14 loci. Linkage disequilibrium was present in our dataset, but the standardized index of association across all loci was small ($\bar{r}$_d_ = 0.032; *p*‐value = 0.001; Figure S3). The mean (± SD) standardized index of association between all pairwise combinations of loci was 0.033 ± 0.035, indicating that most pairs of markers share only a small proportion of variation (Figure S4). The STRUCTURE analysis identified two major genetic clusters, northwest (*n* = 66) and southeast (*n* = 50), which split near the intersection of Highways 1 and 5 (Figures S5–S7). The northwest cluster split into two additional clusters along an east–west gradient, while the southeast cluster split into two additional clusters (Figures S8 and S9).

**FIGURE 2:**
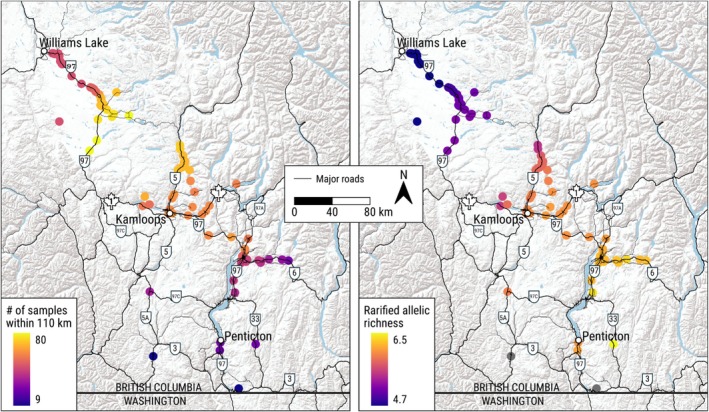
Total number of genetic samples (left) and rarefied (using a minimum sample size of 24) allelic richness (right) within a spatial‐genetic neighborhood of 110 km of each sample from 114 American badgers ( *Taxidea taxus jeffersonii* ) sampled from 2008 to 2023 in southern British Columbia, Canada. Dark gray lines indicate major roads. We did not estimate rarefied allelic richness for the two southernmost samples, shown in gray, because they had too few samples (9 and 10, respectively) within their spatial‐genetic neighborhoods.

Our full dataset of 116 genetic samples produced 6670 pairwise connections. We excluded pairs of samples < 3 km apart to minimize noise from highly clustered sample locations and to help avoid overfitting in resulting predictions. After removing these pairs, our final analysis dataset contained 6514 pairs of genetic samples. Pearson's correlation of genetic distance and (straight‐line) geographic distance for these 6514 genetic sample pairs was 0.264 (95% confidence interval = 0.241–0.286; Figure S10). We observed positive autocorrelation in spatial genetic structure up to ~110 km (Figure S11), which we used as the radius of the genetic neighborhoods for calculating rarefied allelic richness. Rarified allelic richness across all loci was lowest in the northwest (Cariboo region) and increased towards the southeast extent of the study area (Figure 2). We did not calculate rarefied allelic richness for the two spatial genetic neighborhoods that encompassed 9 and 10 genetic sample locations, respectively. The remaining 114 neighborhoods included between 24 and 80 sample locations, so we used a sample size of 24 for rarefication. Ninety‐nine of the 116 samples (85%) were collected within 500 m of a major road.

### 3.2 Landscape Resistance Model Results

**FIGURE 3:**
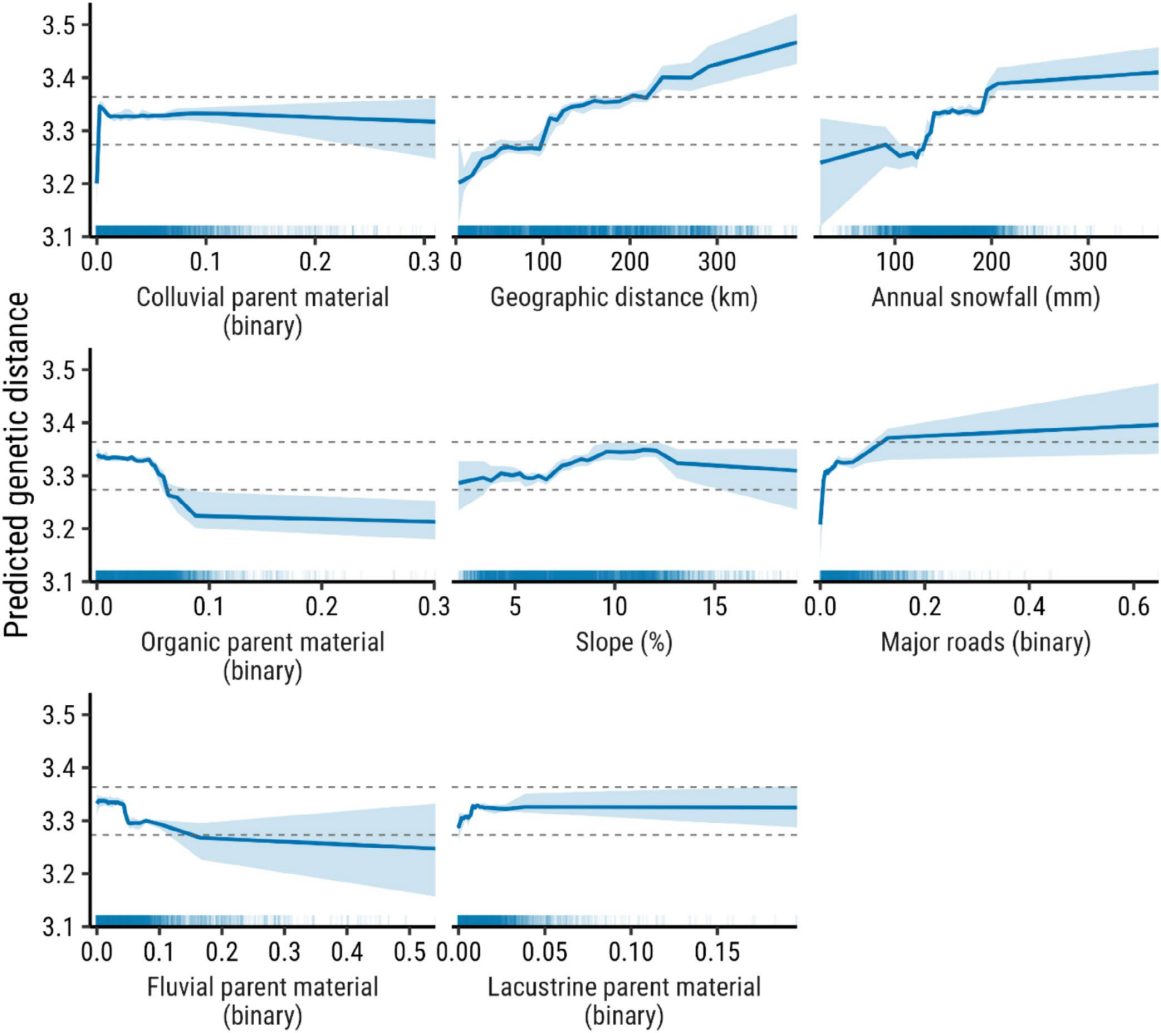
Accumulated local effects (ALE) plots showing the predicted change in Euclidean genetic distance across a range of values for different landscape variables from the top gradient boosting machine model using data from 116 American badgers ( *Taxidea taxus jeffersonii* ) from 2008 to 2023 in southern British Columbia, Canada. Shaded regions represent 95% confidence intervals generated from a model fitted separately to 500 bootstrapped samples. The region between the two dashed lines indicates the range where ALE predictions for 95% of purely random variables lie, meaning that areas where the bootstrapped confidence intervals do not overlap this band are statistically significant (non‐random). Variables for this model were average values extracted along 500‐m buffered straight lines between pairs of genetic sample locations. Rug marks along the *x*‐axis indicate the distribution of values in the dataset for each variable.

**FIGURE 4:**
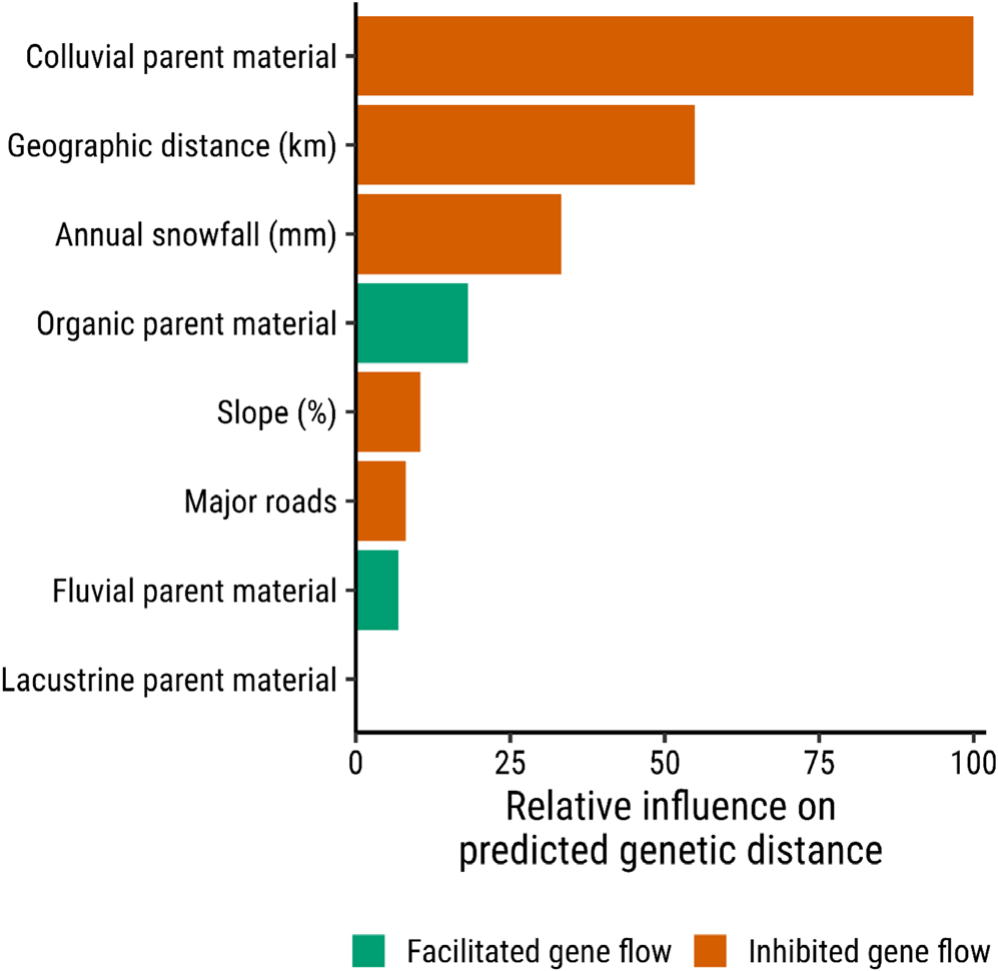
Relative influence of variables predicting pairwise Euclidean genetic distances from the top gradient boosting machine model fit using data from 116 American badgers ( *Taxidea taxus jeffersonii* ) from 2008 to 2023 in southern British Columbia, Canada.

Colluvial parent material between pairwise genetic sample locations inhibited gene flow and had the most influence (relative influence = 100) on variation in predicted genetic distances in our top model (Figures 3 and 4). Steep slopes, increased snowfall, and the presence of major roads were also associated with higher pairwise genetic distance, indicating that these features consistently impede gene flow (Figure 3). Organic parent material facilitated gene flow, while higher annual snowfall inhibited it. Geographic distance was an important predictor of genetic distance with a relative influence of 55 (Figures 3 and 4). Pairwise geographic distances above ~100 km led to increased genetic differentiation from the median genetic distance (Figure 3). Canopy cover, along with several land cover and soil parent material variables, was not retained in variable selection (Figure S12). Our final model retained fewer variables and had smoother response curves than a model with randomly assigned (aspatial) cross‐validation folds (Figure S13).

Pearson's correlation between predicted and observed genetic distances from our top model using the full dataset was 0.429 (95% confidence interval = 0.409–0.449; Figure S14), compared to 0.291 (0.268–0.313) for a model fitted with geographic distance as the only explanatory variable. The six withheld test datasets in the spatial cross‐validation represented 69.5% ± 12.0 (mean ± SD; range = 49.6%–84.8%) of the full dataset. We observed high multicollinearity between slope and other landscape variables retained in the top‐performing straight‐line model, including a positive correlation with colluvial parent material (*r* = 0.87, *p*‐value < 0.001), and a negative correlation with organic parent material (*r* = −0.69, *p*‐value < 0.001; Figure S15). However, slope was retained because it increased the model's predictive capacity (lower RMSE_test_) overall, including in four of six spatial cross‐validation folds. There was a much lower degree of spatial overlap in pairwise straight‐line transects than in LCP transects (across all LCP iterations; Figure S16). We report the optimal combination of hyperparameters (i.e., produced the lowest average RMSE_test_) for GBMs fit using Euclidean genetic distance for each model iteration (straight‐line and five LCP models) in Table 2.

**TABLE 2:** Comparison of variables selected (in order of relative influence), mean RMSE_test_ across six withheld spatial cross validation test folds, R‐squared (using full model), and the optimal set of hyperparameters for each gradient boosting tree model iteration predicting Euclidean genetic distance from geographic distance and landscape characteristics for 116 American badgers ( *Taxidea taxus jeffersonii* ) in southern British Columbia, Canada.

Model iteration	Variables selected	Mean RMSE_test_	*R* ^2^ full model	Number of trees	Learning rate	Min # of observations per node	Interaction depth
Straight‐line	Geographic distance	0.3573	0.0848	250	0.005	50	2
**Straight‐line**	**Colluvial parent material, Geographic distance, Annual snowfall, Organic parent material, Slope, Major roads, Fluvial parent material, Lacustrine parent material**	**0.3431**	**0.1844**	**1710**	**0.005**	**25**	**2**
LCP 1	Colluvial parent material, Geographic distance, Lacustrine parent material, Major roads	0.3439	0.1438	1880	0.005	50	1
LCP 2	Colluvial parent material, Geographic distance, Slope, Organic parent material	0.3453	0.1279	1130	0.005	50	1
LCP 3	Colluvial parent material, Geographic distance, Annual snowfall, Lacustrine parent material, Major roads	0.3435	0.1706	1210	0.005	50	2
LCP 4	Geographic distance, Annual snowfall, Fluvial parent material, Organic parent material	0.3463	0.1396	1660	0.005	5	1
LCP 5	Colluvial parent material, Geographic distance, Organic parent material, Annual snowfall	0.3443	0.1330	910	0.005	25	1

*Note:* A straight‐line model using geographic distance as the only explanatory variable is included for comparison. Bolded values indicate the final model used for spatial predictions of landscape resistance and connectivity models. LCP denotes least‐cost path.

Separate models (without variable selection) fitted to each region suggested that predictions for organic and lacustrine parent materials largely reflected patterns in the Cariboo (northwest) region and conditions between the Cariboo and southeast regions, a pattern mostly driven by conditions within the Cariboo region and those between the Cariboo region and the three southeast regions (Figure S17). The negative effect of major roads on gene flow was strongest in pairwise transects connecting the Cariboo region with the three southeast regions. Apart from geographic distance, landscape variables had relatively little effect on predicted genetic distances within the three southeast regions (Figure S17).

**FIGURE 5:**
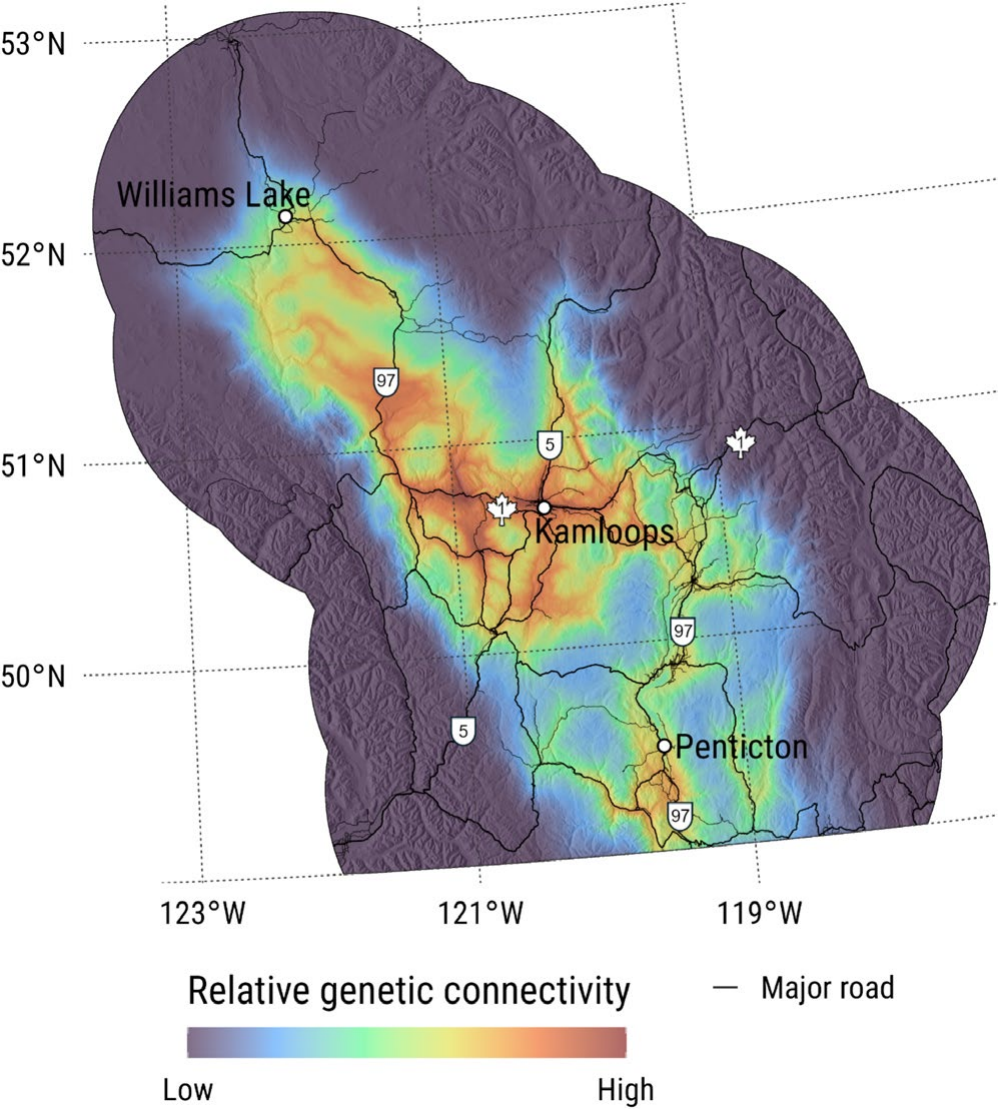
Predicted relative genetic connectivity from resistant kernels for American badgers ( *Taxidea taxus jeffersonii* ) modeled using 116 genetic samples collected from 2008 to 2023 in southern British Columbia. Resistance kernels used landscape resistance as the input layer, which was predicted from a gradient boosting machine model with pairwise Euclidean genetic distances as the response variable and landscape data summarized along straight‐line transects between pairs of genetic samples as explanatory variables. Predictions are plotted over a hillshade layer to highlight topography. Source locations were restricted to the known spatial distribution of the western population of *T. t. jeffersonii*.

Mapped predictions of landscape resistance and genetic connectivity showed areas of relatively low resistance and high connectivity were present in broad swaths of the Cariboo region with organic and fluvial parent materials but were largely confined to valley bottoms within the Thompson, Okanagan, and Nicola regions (Figures 5 and S18). The few corridors with relatively predicted high genetic connectivity between the Thompson and Okanagan regions were located within narrow valleys, such as along Highway 97 southeast of Kamloops (Figure 5). Most areas with the highest predicted genetic connectivity from resistant kernels either paralleled major highways (e.g., Highway 97 immediately southeast of Williams Lake, and Highway 97C south of Kamloops) or crossed them (e.g., Highway 97 near 100 Mile House).

Results from GBM models fitted using different genetic distance metrics as response variables also found that straight‐line models consistently performed better than all subsequent LCP models. The straight‐line model using Euclidean genetic distance as the response variable (our top model overall; adjusted RMSE_test_ = 0.167) outperformed models using the proportion of shared alleles (adjusted RMSE_test_ = 0.176) and the Queller and Goodnight relatedness estimator (adjusted RMSE_test_ = 0.199). The relationships between landscape variables and genetic (dis)similarity were similar for each metric (Figure S19), and their final resistance surfaces were highly correlated (pairwise Pearson's correlations > 0.85; Table S4).

## 4 Discussion

We found strong evidence that landscape attributes influenced the spatial genetic structure of *T. t. jeffersonii* in BC. As hypothesized, human development (major roads), topography, and soil parent material influenced genetic connectivity. Gene flow was strongly impeded by colluvial soil parent material, which was highly correlated with steep slopes and is strongly avoided by badgers in southern BC (Apps et al. 2002; Kinley et al. 2013). Fluvial parent material facilitated gene flow yet was not a significant predictor of badger habitat selection elsewhere in southern BC (Apps et al. 2002; Kinley et al. 2013). Our finding that steep slopes confer high landscape resistance for badgers is consistent with results from previous studies showing avoidance of these areas (Apps et al. 2002; Kinley et al. 2013). However, in other parts of their range, badgers select intermediate to steep slopes, especially at finer spatial scales (e.g., den placement; Quinn 2008; Sunga et al. 2017). The retention of slope in our models likely reflects the increased energy expenditure required to cross steep terrain (e.g., mountains and steep river canyons) at larger spatial scales. The convergence of results across the three genetic distance metrics suggested that our choice of individual‐based metric did not influence our overall inference. Our hypotheses that land cover (grassland, shrubland, wetland) and canopy cover would influence badger genetic connectivity were not supported, as none of these variables were retained in the final model.

Major roads were an important impediment to gene flow for *T. t. jeffersonii*, corroborating results from Ford (2017), despite these roads having smaller footprints than other landscape variables in the model. Long, linear valley bottoms with friable soils, low snowfall, and flat terrain serve as natural movement corridors, but may increase mortality risk by funneling badgers into areas bisected by highways and other high‐traffic roads. The increase in predicted landscape resistance from a pairwise transect intersecting at least one major road was roughly equivalent to the predicted resistance offered by 100 km of geographic distance (Figure 3). The negative effect of roads on gene flow in *T. t. jeffersonii* is consistent with those from landscape genetics analyses in peripheral populations of woodland caribou ( *Rangifer tarandus caribou* ; Anderson and Thomson 2024) and fisher ( *Pekania pennanti* ; Tucker et al. 2017). Coupled with existing data on highway mortalities, our map of predicted genetic connectivity can inform efforts to increase road permeability for badgers by targeting areas for new wildlife‐specific underpasses and fencing construction, modification of existing underpasses, and placement of road signs to increase driver awareness of important badger crossing areas.

We predicted relatively high genetic connectivity within much of the Cariboo region despite the high road mortality documented in this area (Klafki 2014). Besides major roads, the Cariboo region was largely devoid of colluvial parent material and had relatively few steep slopes, two major factors that were predicted to impede gene flow. In contrast, these two landscape attributes were ubiquitous in the Okanagan region, and areas with low predicted landscape resistance were heavily concentrated in a few linear valleys with a high density of major roads. These few corridors likely represent areas of high conservation value whose protection would help maintain genetic connectivity in the region.

We found that genetic connectivity in the western population of *T. t. jeffersonii* generally aligned with their habitat preferences, suggesting that landscape attributes likely contribute more to badger spatial genetic structure in southern BC than dispersal capability. Across a much larger spatial extent that did not include peripheral populations, Kierepka and Latch (2016a) found that the high dispersal capability of badgers overrode the influence of habitat preferences so that large water bodies and isolation by distance were the only main drivers of their spatial genetic structure across North America. In a later study, the same authors found that isolation by distance remained the strongest predictor of badger genetic differentiation at a finer spatial scale in Wisconsin. Moreover, they speculated that badgers successfully dispersed across suboptimal conditions (e.g., cultivated areas), despite avoiding them in many parts of their range (Kierepka and Latch 2016b). The relatively strong influence of landscape attributes on badger genetic differentiation in our study may reflect the steep gradients in slope and snowfall in the southern portion of BC, which were also highly correlated with the spatial distribution of roads and soil parent materials.

We speculate that our genetic sample distribution (i.e., spatially clustered locations separated by large expanses devoid of samples) may have contributed to the straight‐line model outperforming all LCP models, a finding that is contrary to results from past studies using this iterative corridor‐based approach in other species (Pless et al. 2021; Bishop et al. 2021; Palm et al. 2023). Pairwise LCPs connecting sample locations often converged into a single transect, resulting in a high degree of spatial overlap (Figure S16). Notably, over half of the total number of LCP transects that connected genetic samples from the Cariboo region to those in any other region followed the exact same ~130 km long corridor running from 70 Mile House south through Cache Creek and east to Kamloops in the Thompson region, highlighting a potentially important movement corridor. This overlap greatly reduced the amount of variation in the extracted landscape variables used to predict genetic distance in the LCP models compared to the straight‐line model, possibly resulting in poorer model performance.

Sampling design and concomitant spatial autocorrelation in landscape genetics studies can bias results and confound inference by highlighting certain features perceived to influence gene flow that may instead reflect the sampling configuration itself (Schwartz and McKelvey 2009). Because most genetic samples were collected opportunistically from road mortalities, our results may be biased towards male badgers, whose larger home ranges and longer dispersal distances compared to females make them more likely to encounter roads (Thomas et al. 2022). We attempted to account for the high spatial autocorrelation in our data and reduce overfitting by incorporating spatial cross validation and variable selection into our GBM models (Meyer et al. 2019). The smoother response curves from our top model compared to those from a model with randomly assigned cross‐validation folds suggested that spatial cross‐validation may have reduced overfitting and yielded more generalizable predictions. Our additional analysis of separate models fitted within regions found higher uncertainty around predictions of landscape resistance and genetic connectivity in the far southeastern portions of our study area (i.e., Nicola and southern Okanagan regions) due to lower sample sizes in these areas.

Future genetic sampling of badgers away from major roads, coupled with simulations (*sensu* Oyler‐McCance et al. 2013), would clarify how sample distribution influences variable selection, model performance, and overall inference in an iterative, transect‐ and individual‐based modeling framework. Increased genetic sampling in the southern portion of our study area, along with southeast BC's Kootenay region and southwest Alberta, would help improve our genetic connectivity predictions and identify potential movement corridors between the western and eastern *T. t. jeffersonii* populations. Furthermore, incorporating samples from northeast Washington could help identify and protect movement corridors that connect to larger, more contiguous swaths of habitat and populations with higher genetic variation (Ford et al. 2019), thereby promoting persistence of this peripheral population.

We observed increasing isolation by distance and allelic richness moving from the northwest towards the southeast, consistent with the central‐marginal hypothesis (Eckert et al. 2008) and results in Ford et al. (2019). Furthermore, our STRUCTURE analysis found that badgers in the Thompson, Okanagan, and Nicola regions in the southeast likely composed one genetic cluster separate from those in the Cariboo region in the northwest, consistent with STRUCTURE results in Ford et al. (2019). Together, these results underscore the need to preserve genetic connectivity between the northwest and southeast portions of the study area to maintain the genetic diversity of badgers in the Cariboo region.

Conserving and connecting genetically distinct peripheral populations are often high priorities for preserving genetic diversity, resilience, and biodiversity, especially in the face of increasing global change (DeWoody et al. 2021). In this study, we identify landscape attributes influencing genetic connectivity for an endangered, peripheral carnivore population and highlight potential areas where management and conservation efforts to maintain or restore connectivity may be most effective. Specifically, our results underscore the need to improve road permeability for peripheral populations, which may be more susceptible to the negative effects of impeded genetic connectivity (Row et al. 2016).

## Author Contributions

**Eric C. Palm:** conceptualization (equal), formal analysis (lead), investigation (equal), methodology (lead), writing – original draft (lead), writing – review and editing (equal). **Erin L. Landguth:** conceptualization (equal), formal analysis (supporting), investigation (equal), methodology (supporting), writing – original draft (supporting), writing – review and editing (equal). **Karina Lamy:** conceptualization (equal), data curation (supporting), methodology (supporting), project administration (supporting), writing – original draft (supporting), writing – review and editing (equal). **Jamieson C. Gorrell:** data curation (lead), formal analysis (supporting), methodology (supporting), writing – review and editing (equal). **Richard D. Weir:** data curation (supporting), writing – review and editing (equal). **Emma L. Richardson:** data curation (supporting), formal analysis (supporting), writing – review and editing (supporting). **Krystyn J. Forbes:** data curation (supporting), writing – review and editing (supporting). **Helen Davis:** data curation (supporting), writing – review and editing (supporting). **Joanna M. Burgar:** conceptualization (equal), data curation (supporting), project administration (lead), supervision (lead), writing – original draft (supporting), writing – review and editing (supporting).

## Funding

This work was supported by Habitat Conservation Trust Foundation and Environment and Climate Change Canada.

## Disclosure

Benefit‐sharing statement: Benefits from this research accrue from the sharing of our code and results on public repositories as described above.

## Conflicts of Interest

The authors declare no conflicts of interest.

## Supporting information


**Appendix S1:** ece373467‐sup‐0001‐AppendixS1.docx.

## Data Availability

Data and code to fit straight‐line and least‐cost path gradient boosting machines, and predict landscape resistance and connectivity, along with code to extract variables along straight lines and least‐cost paths between location pairs, are publicly available at https://doi.org/10.5281/zenodo.17885808. This repository does not include actual GPS locations for genetic samples, yet the included scripts allow full reproduction of analyses. Individual genotypes with geomasked sample locations that were used in the analyses are available from the corresponding author by request, subject to approval from the British Columbia Ministry of Water, Land and Resource Stewardship.
