# Supplementary material for: Roads, Soil, Snow, and Topography Influence Genetic Connectivity: A Machine Learning Approach for a Peripheral American Badger Population
Source: Ecol Evol. 2026 Apr 16;16(4):e73467. doi: 10.1002/ece3.73467 (PMC13086617; doi:10.1002/ece3.73467)
Supplement: Supplementary file 1 — Appendix S1: ece373467‐sup‐0001‐AppendixS1.docx. [file ECE3-16-e73467-s001.docx]

**Supporting Information for:**

*Roads, soil, snow, and topography influence genetic connectivity: A machine learning approach for a peripheral American badger population*

Journal: *Ecology and Evolution*

Article DOI: <https://doi.org/10.1002/ece3.73467>

Eric C. Palm, Erin L. Landguth, Karina Lamy, Jamieson C. Gorrell, Richard D. Weir, Emma L. Richardson, Krystyn J. Forbes, Helen Davis, Joanna M. Burgar

This file includes:

Tables S1–S3

Figures S1–S19

Table S1. Numbers of American badger (*Taxidea taxus jeffersonii*) genetic samples analyzed in this study including samples that were reanalyzed from Ford et al. 2019.

| Region | This study | Ford et al. 2019 |
| --- | --- | --- |
| Cariboo | 48 | 37 |
| Thompson | 40 | 12 |
| Okanagan | 26 | 13 |
| Nicola | 2 | 2 |
| Total | 116 | 64 |

Table S2. Microsatellite loci for 116 American badgers (*Taxidea taxus jeffersonii*) from southern British Columbia, Canada, including primer sequence, PCR annealing temperature (*Ta*), number of alleles (*k*), observed heterozygosity (*H_O_*), unbiased expected heterozygosity (*uH_E_*), and Wright’s fixation index (*F_IS_*). Pigtails added to reverse primers are underlined.

| Locus | Forward (5’ – 3’) | Reverse (5’ – 3’) | *Ta (ºC)* | *k* | *H_O_* | *uH_E_* | *F_IS_* |
| --- | --- | --- | --- | --- | --- | --- | --- |
| Gg234 | TTACTTAGAGGATGATAACTTG | GTTTGAACTCATAGGACTGATAGC | 50 | 5 | 0.629 | 0.666 | 0.051 |
| Gg443 | GATCATGTTTGCAATTAAATGT | GTTTGATCCTCCGGTAACTGTTGT | 54 | 10 | 0.647 | 0.728 | 0.108 |
| Gg465 | GATCTTCACAAACAAGCTTC | GTTTGATCTCCTTTCCTCTCTTTG | 55 | 7 | 0.609 | 0.760 | 0.196 |
| Ma-1 | ATTTTATGTGCCTGGGTCTA | GTTTATGCGTCTCTGTTTGTCA | 54 | 7 | 0.621 | 0.614 | -0.015 |
| Mvis072 | CTGCAAAGCTTAGGAATGGAGA | GTTTCCACTACACTGGAGTTTCAGCA | 55 | 6 | 0.679 | 0.690 | 0.011 |
| Tt-1 | AACGGCTTCTAACCACTCCA | GTTTCCCCGCTTTTCATTTCTTTA | 55 | 8 | 0.696 | 0.697 | -0.002 |
| Tt-2 | AGCCAAGACACAGAAACAAC | GTTTCAAGGATTCAAGGACCAT | 57 | 4 | 0.617 | 0.651 | 0.047 |
| Tt-3 | AGTCTGCTTGGGATTTTCTC | GTTTTGCTCTATGATTACACC | 54 | 7 | 0.638 | 0.670 | 0.044 |
| Tt-4 | GGTGAGACCCTGGAAATAGAAA | GTTTGCTAACCAAACCTAGCAATGAT | 58 | 8 | 0.724 | 0.796 | 0.086 |
| Tt13 | CAGCATTGGGCAACTGGAC | GTTTCCATAACTAGAGGTACTTGAAC | 57 | 11 | 0.853 | 0.858 | 0.001 |
| Tt15 | TGAGAAGCCTGACTTGGAGCA | GTTTGAGGTTGTCTGCAACTAGCACTC | 55 | 10 | 0.716 | 0.765 | 0.061 |
| Tt20 | TGCAGCCAAACAGTAACGAC | GTTTGTCGACATGTATGCTGCATGG | 58 | 7 | 0.613 | 0.799 | 0.229 |
| Tt21 | TACCAAACTTGTATCCCATGTAG | GTTTGGTCACCAAGATACGAATGAG | 58 | 7 | 0.757 | 0.776 | 0.021 |
| Tt27 | AGAGTCCAGATATCCATTGACAG | GTTTACATCATTGCAAATACAACAGTTC | 57 | 6 | 0.698 | 0.741 | 0.053 |

Table S3. Results, shown by locus, of exact tests for deviations from Hardy-Weinberg equilibrium with 1000 Monte Carlo permutations across all 14 loci for 116 American badgers (*Taxidea taxus jeffersonii*) in southern British Columbia, Canada. P-values < 0.05 indicate significant deviation from Hardy-Weinberg equilibrium.

| Locus | Chi^2^ | df | p-value |
| --- | --- | --- | --- |
| Gg234 | 8.7 | 10 | 0.444 |
| Gg443 | 158.2 | 45 | 0.015 |
| Gg465 | 262.5 | 21 | 0.000 |
| Ma1 | 95.0 | 21 | 0.072 |
| Mvis072 | 16.8 | 15 | 0.151 |
| Tt1 | 40.4 | 28 | 0.394 |
| Tt2 | 2.5 | 6 | 0.817 |
| Tt3 | 42.1 | 21 | 0.074 |
| Tt4 | 25.8 | 28 | 0.393 |
| Tt13 | 76.0 | 55 | 0.000 |
| Tt15 | 34.8 | 45 | 0.410 |
| Tt20 | 61.5 | 21 | 0.000 |
| Tt21 | 44.8 | 21 | 0.006 |
| Tt27 | 33.9 | 15 | 0.016 |

Table S4. Matrix of pairwise Pearson’s correlation coefficients for landscape resistance surfaces predicted from straight-line models fitted using different genetic distance metrics for 116 American badgers (*Taxidea taxus jeffersonii*) in southern British Columbia, Canada.

|  | Euclidean genetic distance | 1 − Proportion of shared alleles |
| --- | --- | --- |
| Euclidean genetic distance |  |  |
| 1 − Proportion of shared alleles | 0.90 |  |
| 1 – Queller-Goodnight relatedness | 0.88 | 0.85 |


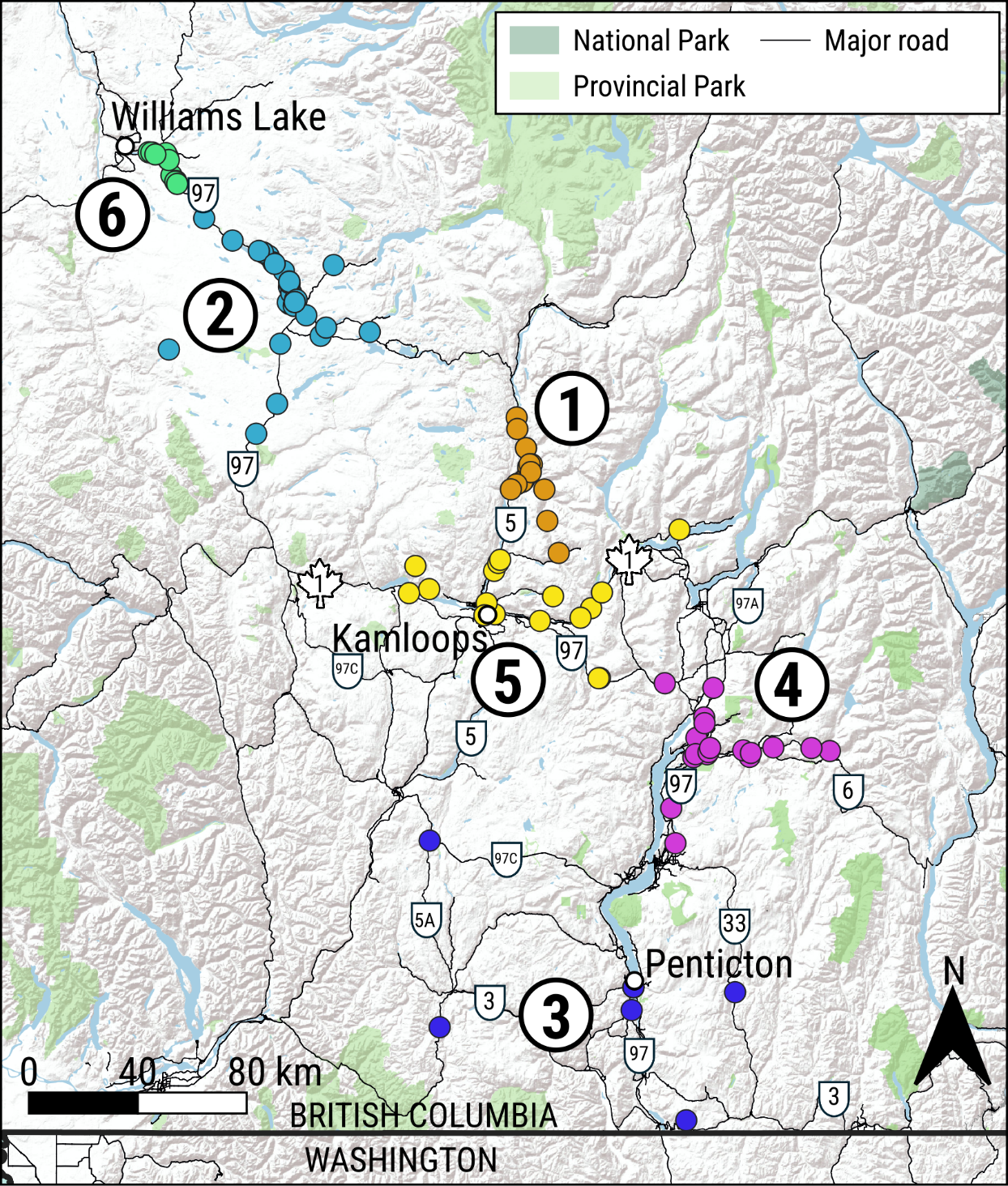


Figure S1. Six spatial clusters used in the leave-one-cluster-out cross validation procedure for gradient boosting tree models predicting genetic distance from geographic distance and landscape characteristics for 116 American badgers (*Taxidea taxus jeffersonii*) in southern British Columbia, Canada.


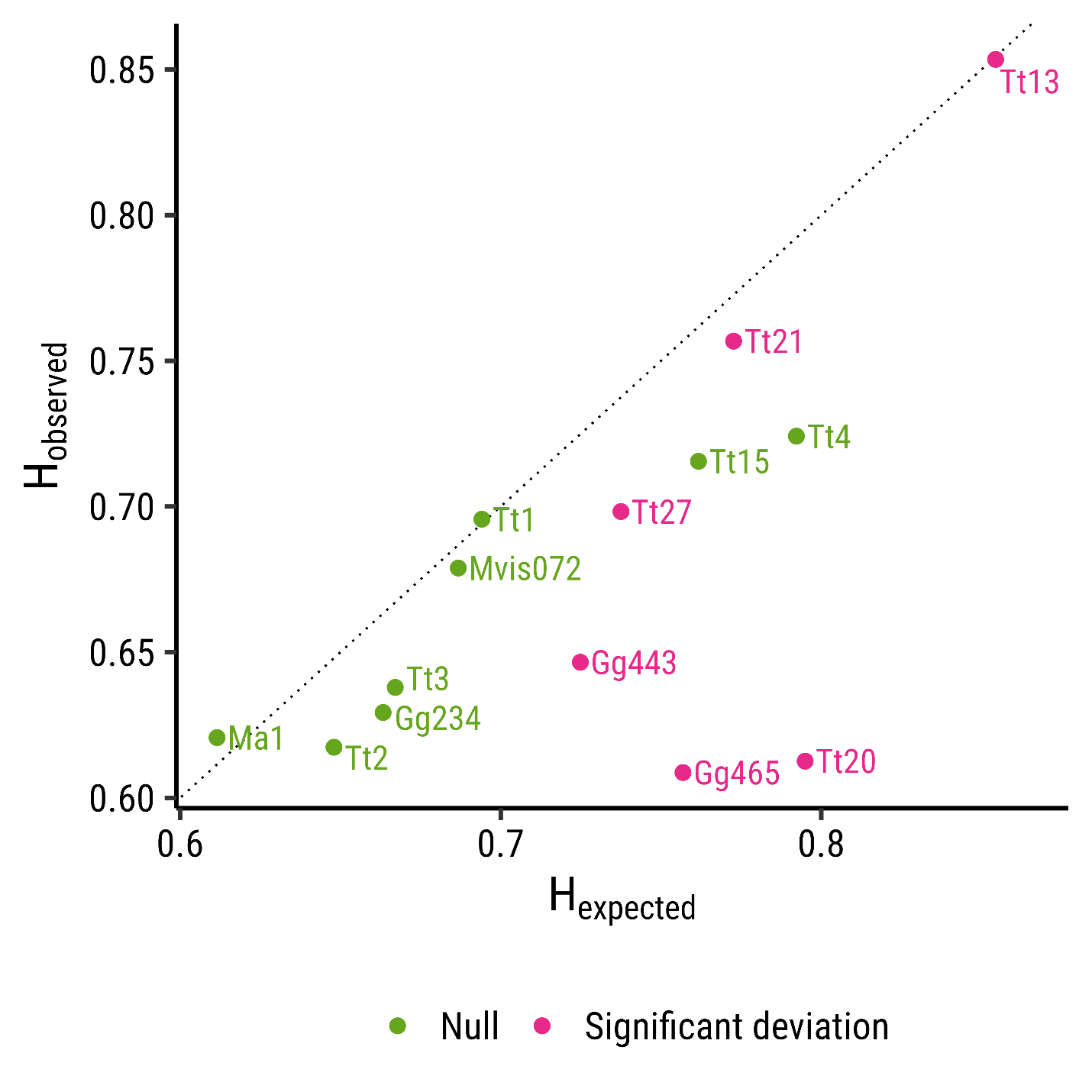

Figure S2. Observed versus expected heterozygosity across 14 loci from 116 American badger (*Taxidea taxus jeffersonii*) genetic samples collected from 2008 to 2023 in southern British Columbia, Canada.


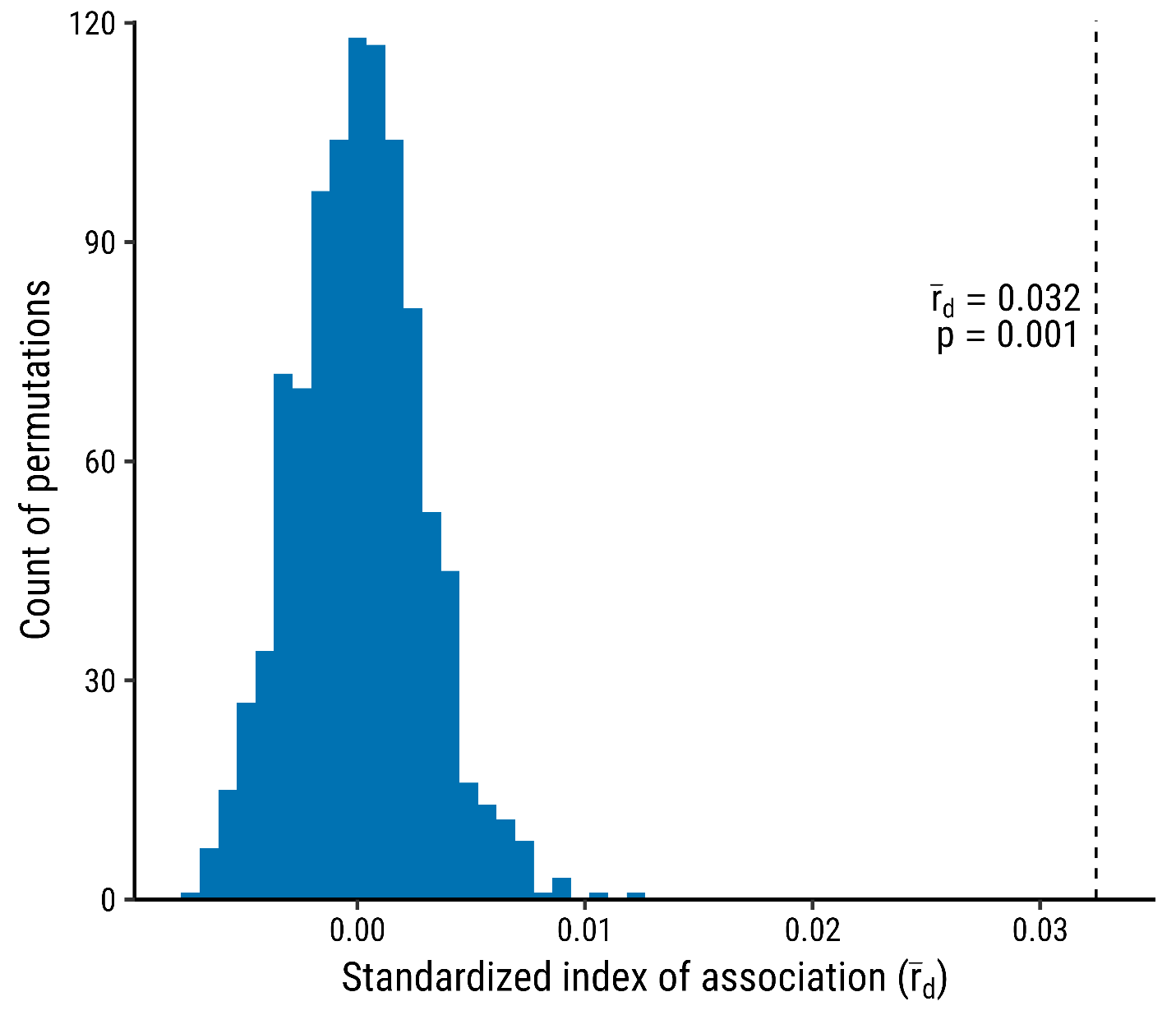


Figure S3. Standardized index of association (r_d_) values across all 14 loci from 116 American badger (*Taxidea taxus jeffersonii*) genetic samples collected from 2008 to 2023 in southern British Columbia, Canada. Higher values of r_d_ and p-values < 0.05 indicate that alleles from different loci occur together at higher-than-expected frequencies.


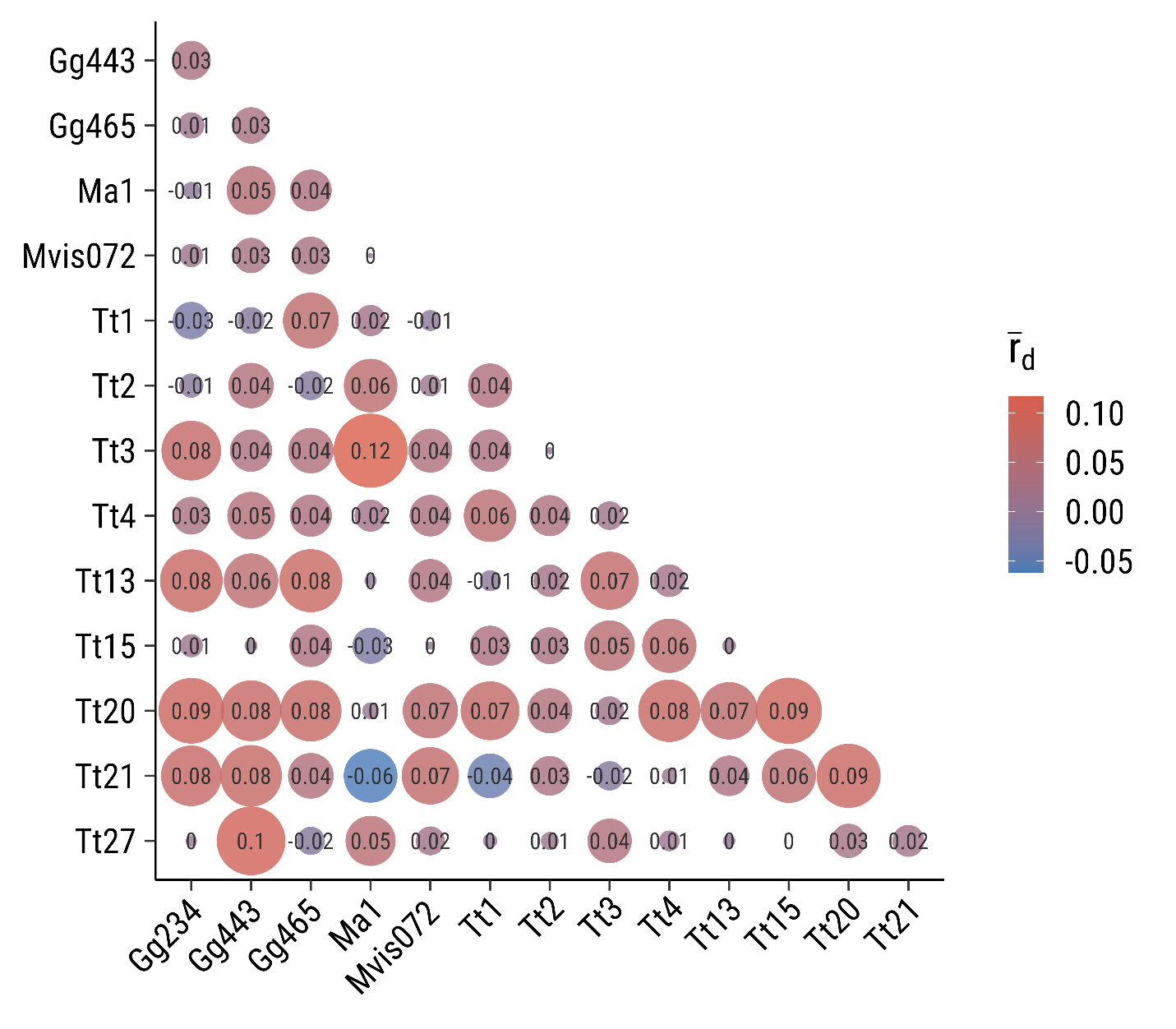


Figure S4. Standardized index of association values for all pairs of 14 loci from 116 American badger (*Taxidea taxus jeffersonii*) genetic samples collected from 2008 to 2023 in southern British Columbia, Canada. Higher values of r_d_ and p-values < 0.05 indicate that alleles from different loci occur together at higher-than-expected frequencies.
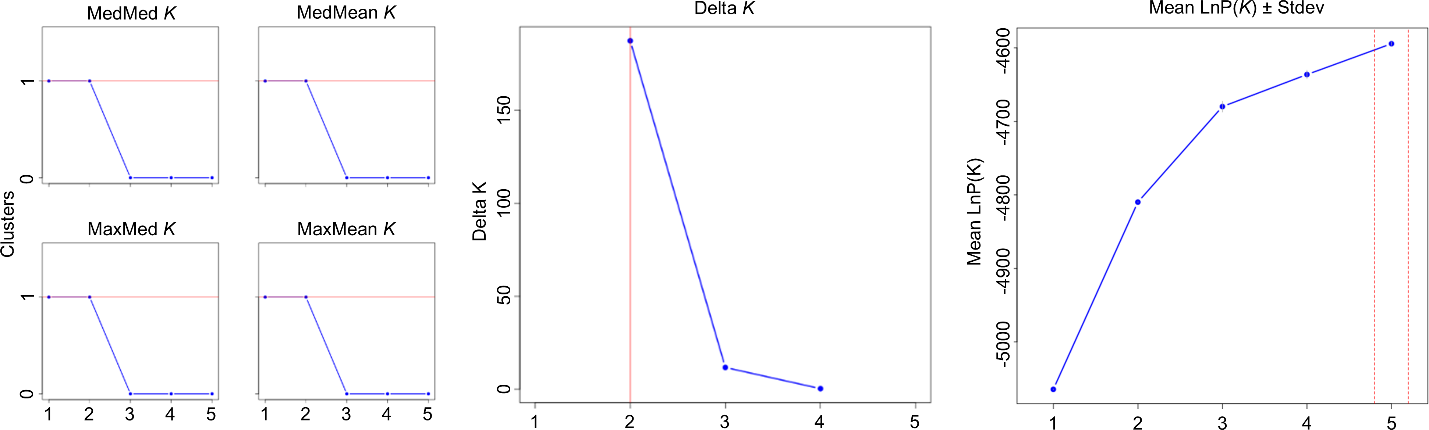


Figure S5. Plots from StructureSelector indicating the optimal number of genetic clusters (*K*) for 116 American badgers (*Taxidea taxus jeffersonii*) whose genetic samples were collected from 2008 to 2023 in southern British Columbia, Canada. The four-panel plot on the left shows the Puechmaille estimators for each possible value of K. Delta *K* shows the change in likelihood between each possible *K*, while the mean LnP(*K*) shows the log probability of each *K.*
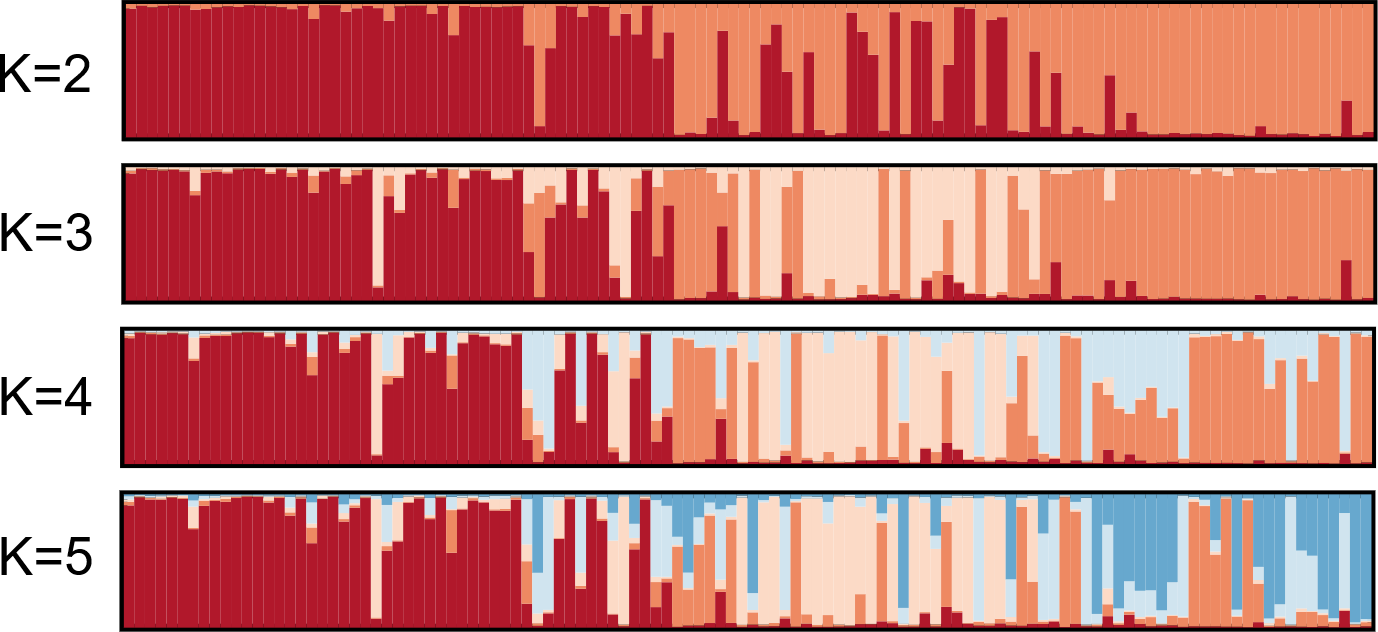
Figure S6.

 STRUCTURE admixture, arranged from west (left) to east (right), for 116 American badgers (*Taxidea taxus jeffersonii*) whose genetic samples were collected from 2008 to 2023 in southern British Columbia, Canada.
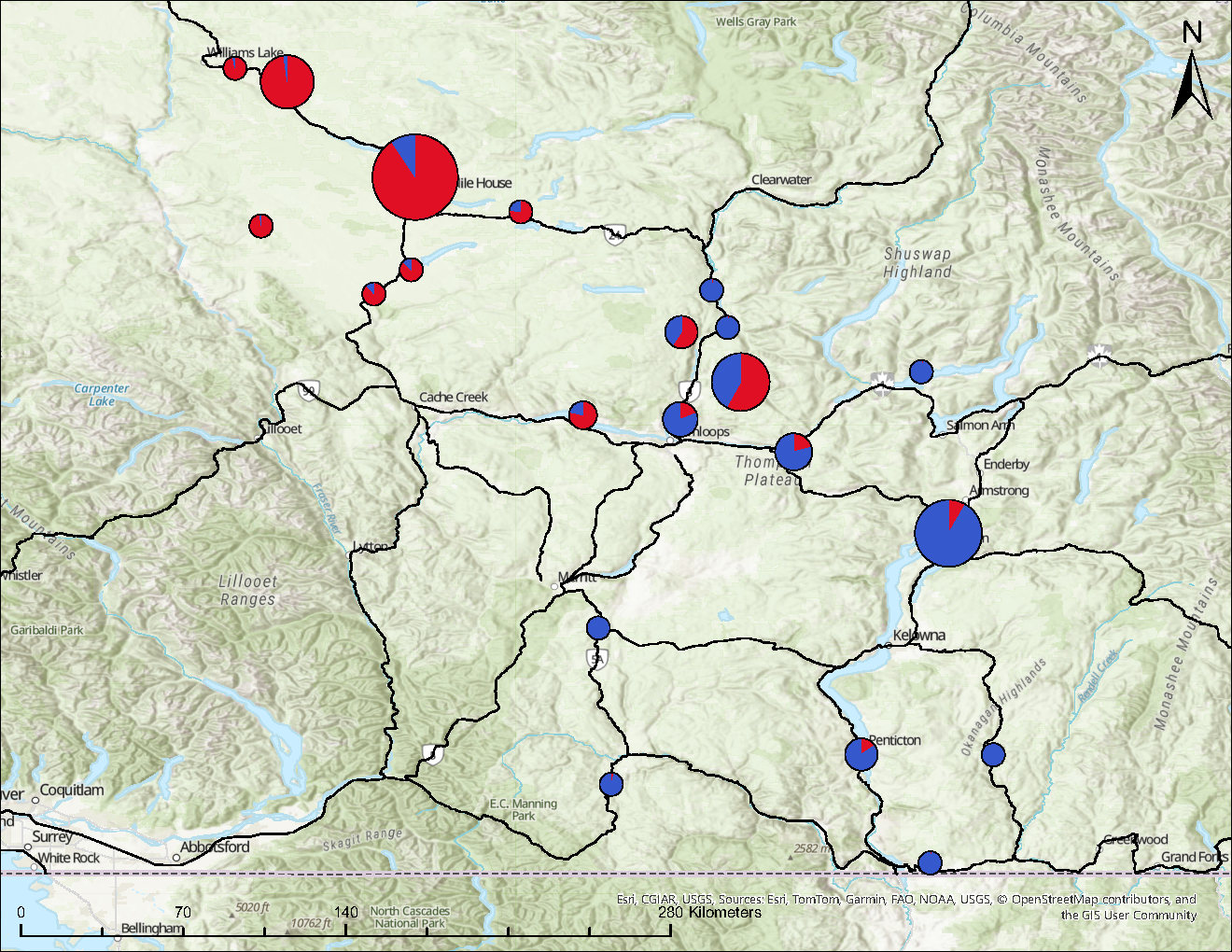


Figure S7. Mapped results of STRUCTURE admixture at K=2 for 116 American badgers (*Taxidea taxus jeffersonii*), whose genetic samples were collected from 2008 to 2023. Clusters separate along Highways 1 and 5 near Kamloops. Larger circles represent more samples.
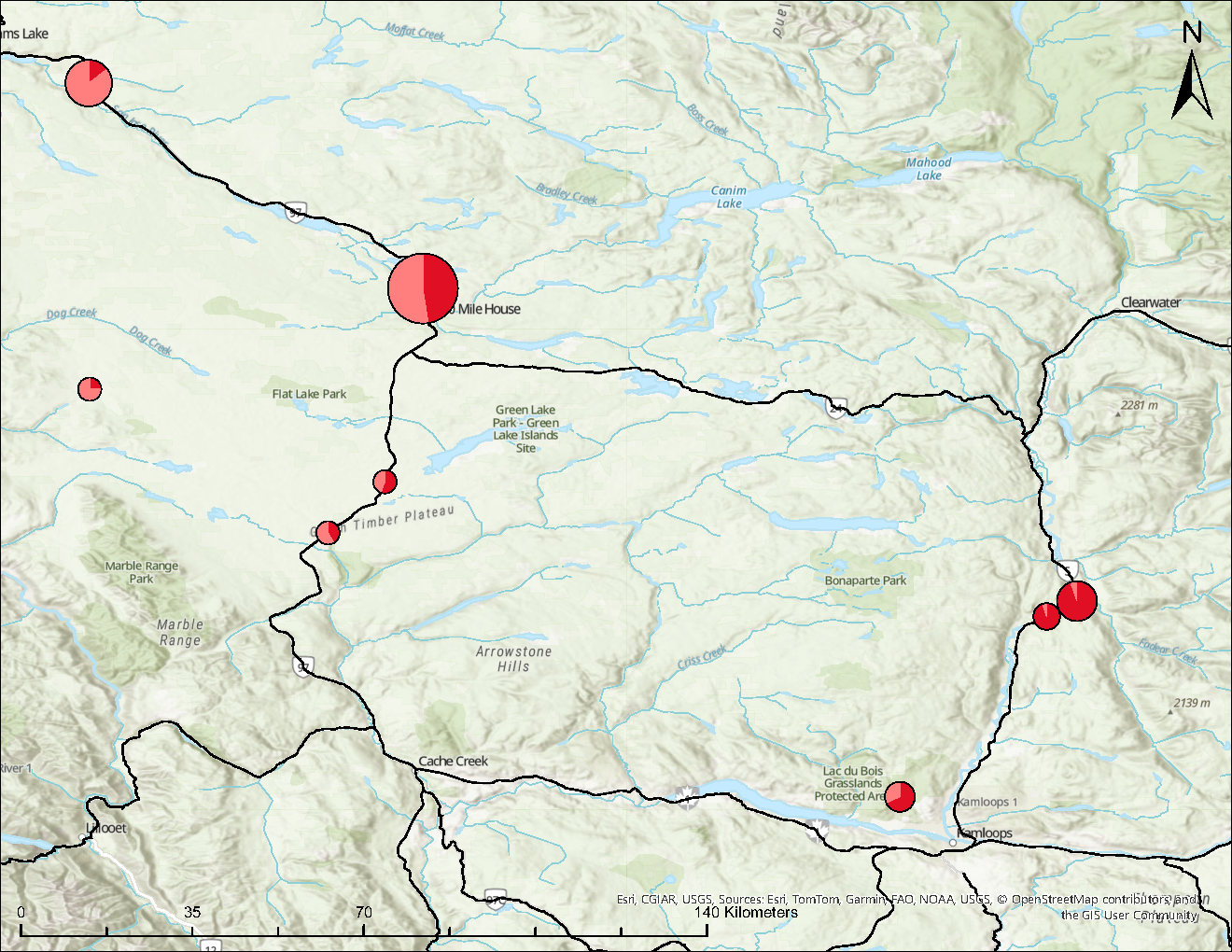
Figure S8. Mapped results of STRUCTURE admixture from the northwest cluster, splitting a second time at K=2, for 66 American badgers (*Taxidea taxus jeffersonii*), whose genetic samples were collected from 2008 to 2023. Larger circles represent more samples.
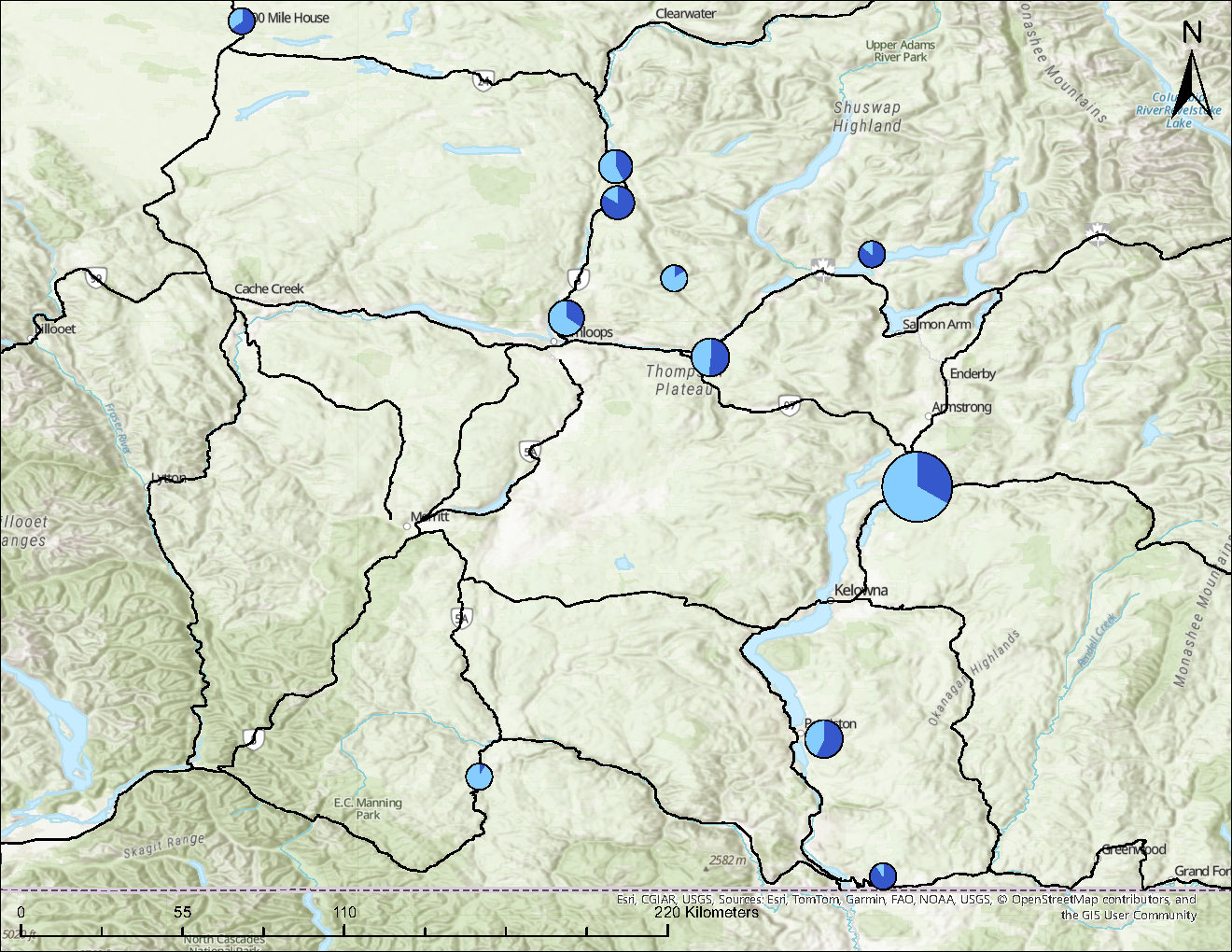
Figure S9. Mapped results of STRUCTURE admixture from the southeast cluster, splitting a second time at K=2, for 50 American badgers (*Taxidea taxus jeffersonii*), whose genetic samples were collected from 2008 to 2023. Larger circles represent more samples.


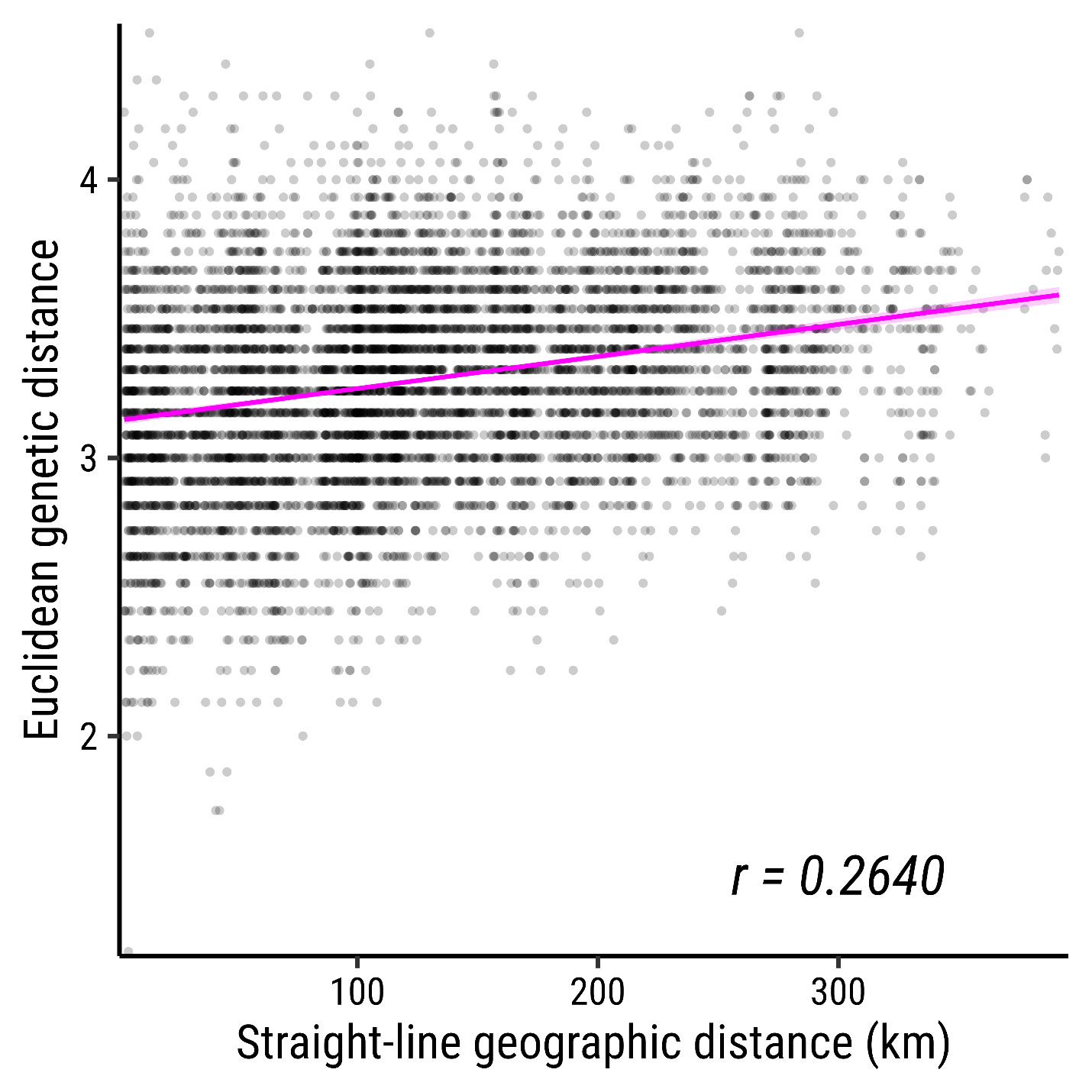


Figure S10. Scatterplot showing the relationship and Pearson’s correlation between Euclidean genetic and straight-line geographic distance for all possible pairwise combinations (n = 6,514; excludes samples with straight-line geographic distance <3 km) of genetic samples from 116 American badgers (*Taxidea taxus jeffersonii*) collected from 2008 to 2023 in southern British Columbia, Canada.


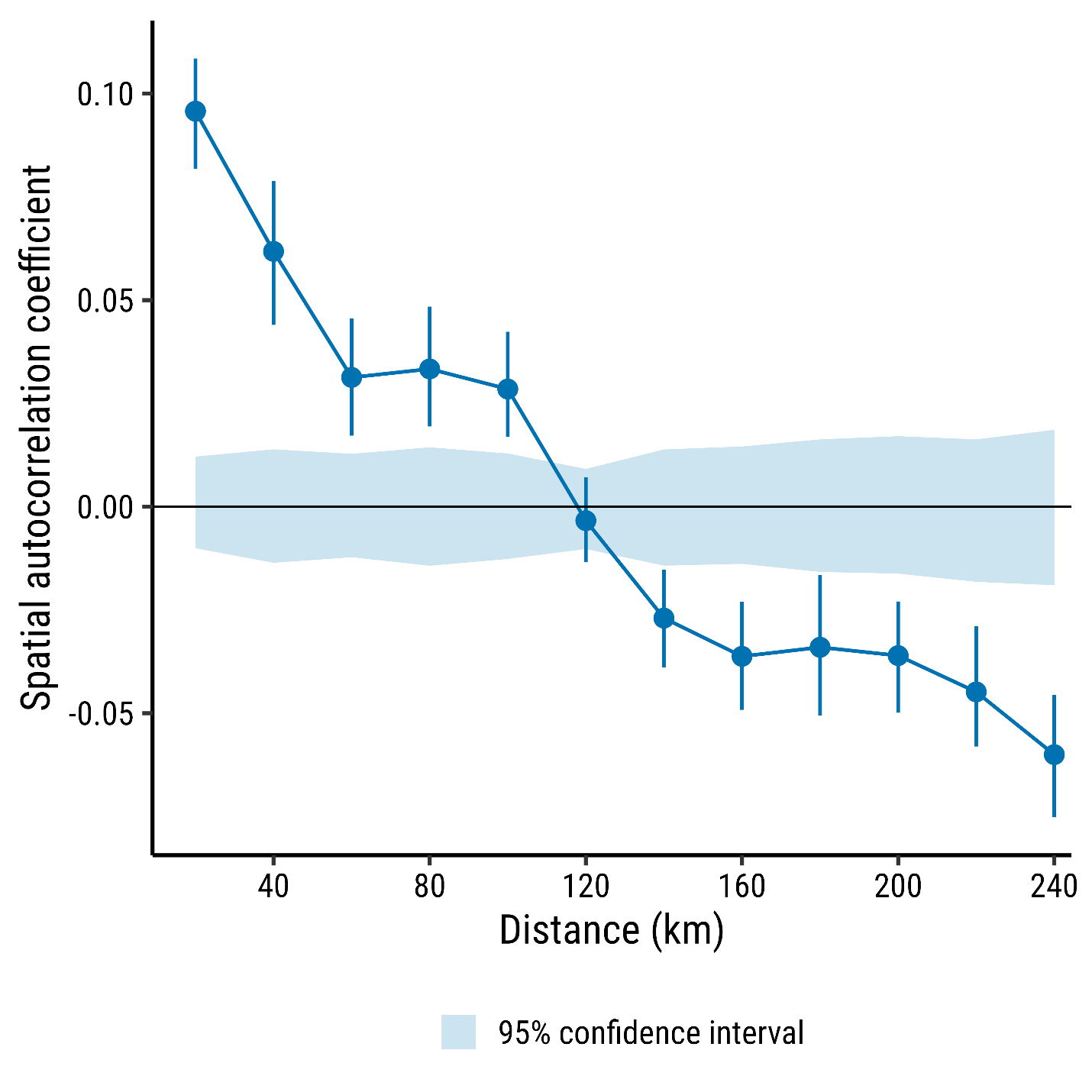


Figure S11. Spatial autocorrelation correlogram for genetic distances for 116 American badgers (*Taxidea taxus jeffersonii*) with genetic samples collected from 2008 to 2023 in southern British Columbia, Canada using the Mantel test statistic. Error bars around the spatial autocorrelation coefficient were determined by bootstrap resampling (999 permutations). Shaded region indicates the 95% confidence interval under the null hypothesis of no autocorrelation between genetic and spatial distances (999 permutations).


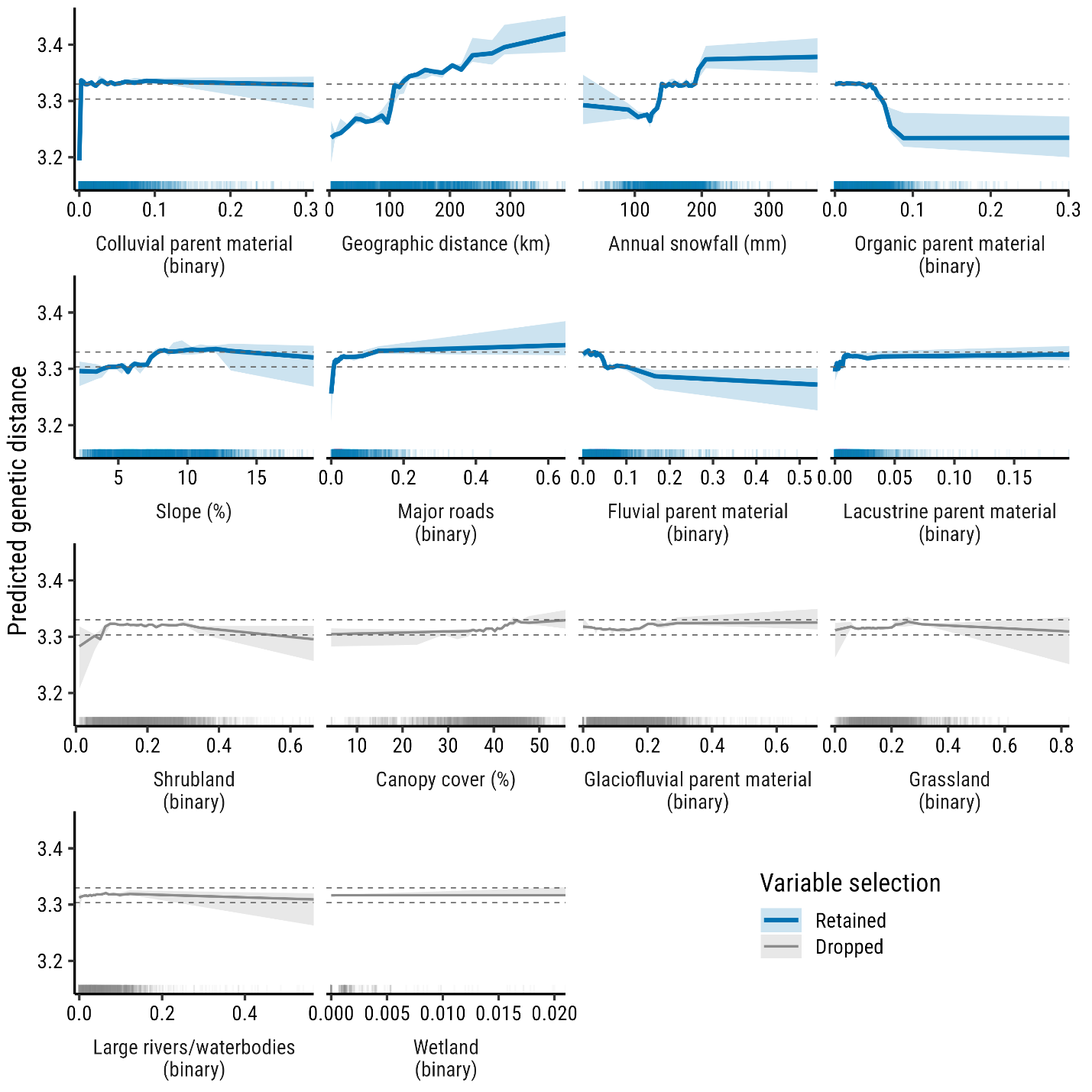


Figure S12. Accumulated local effect plots (ALE) showing the predicted Euclidean genetic distance across a range of values for all landscape variables (i.e., from a model fitted using all landscape variables, without variable selection) hypothesized to influence genetic connectivity from a gradient boosting model using data from 116 American badgers (*Taxidea taxus jeffersonii*) from 2008 to 2023 in southern British Columbia, Canada. Variables that were retained in the final model are depicted in blue. Shaded regions represent 95% confidence intervals generated from the model fitted separately to 500 bootstrapped samples. The region between the two dashed lines indicates the range where ALE predictions for 95% of purely random variables lie, meaning that areas where the bootstrapped confidence intervals do not overlap this band are statistically significant (non-random). Covariates for this model were average values extracted along 500-m buffered straight lines between pairs of genetic sample locations. Rug marks along the x-axis indicate distribution of values in the dataset for each covariate.


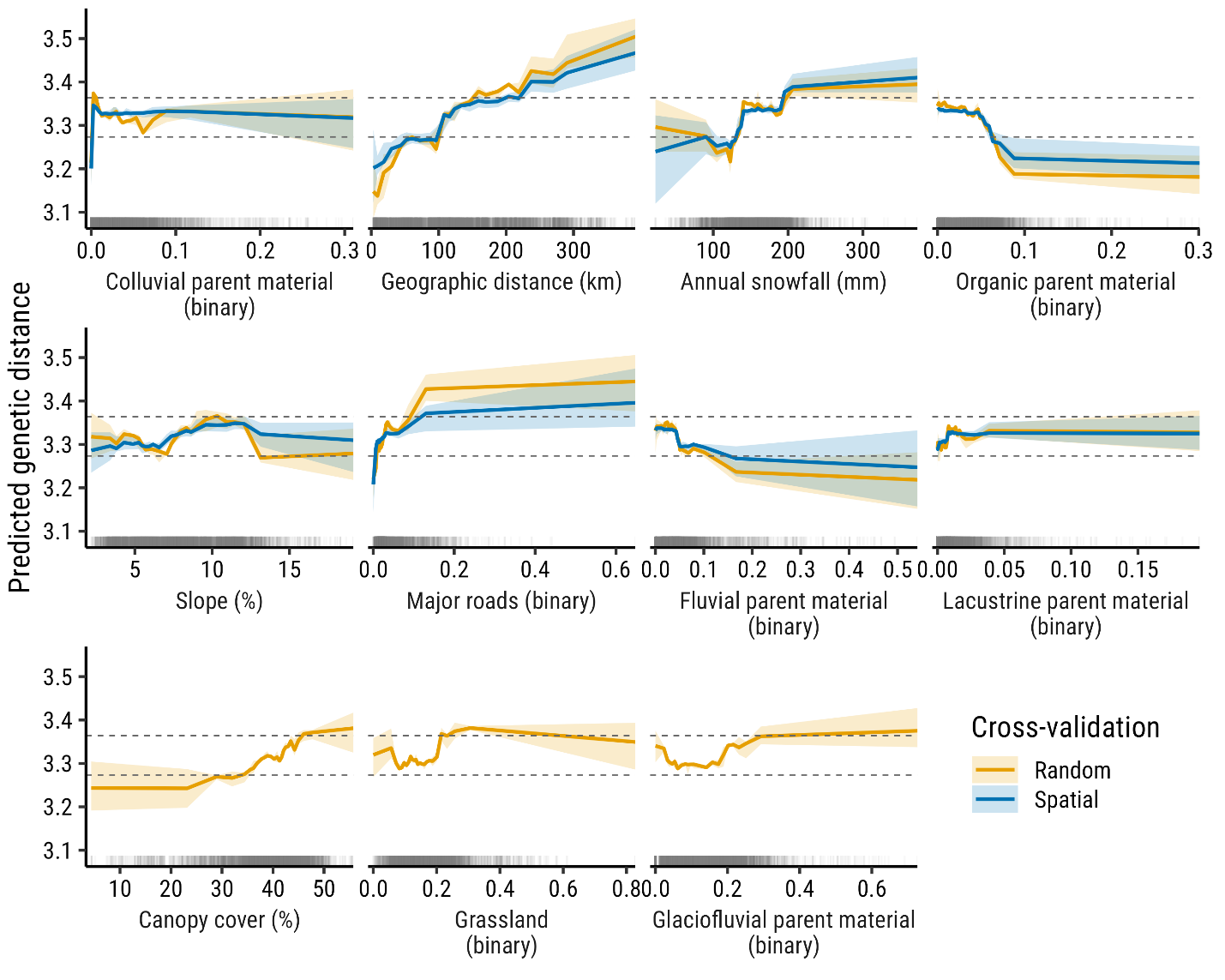
Figure S13. Accumulated local effects (ALE) plots showing the predicted Euclidean genetic distance across a range of values for landscape variables (only those retained in variable selection) influencing genetic connectivity from gradient boosting models fitted using data from 116 American badgers (*Taxidea taxus jeffersonii*) from 2008 to 2023 in southern British Columbia, Canada. Models were fit using six random (aspatial) or spatial folds. Shaded regions represent 95% confidence intervals generated from the models fitted separately to 500 bootstrapped samples. The region between the two dashed lines indicates the range where ALE predictions for 95% of purely random variables lie, meaning that areas where the bootstrapped confidence intervals do not overlap this band are statistically significant (non-random). Covariates for this model were average values extracted along 500-m buffered straight lines between pairs of genetic sample locations. Rug marks along the x-axis indicate distribution of values in the dataset for each covariate.


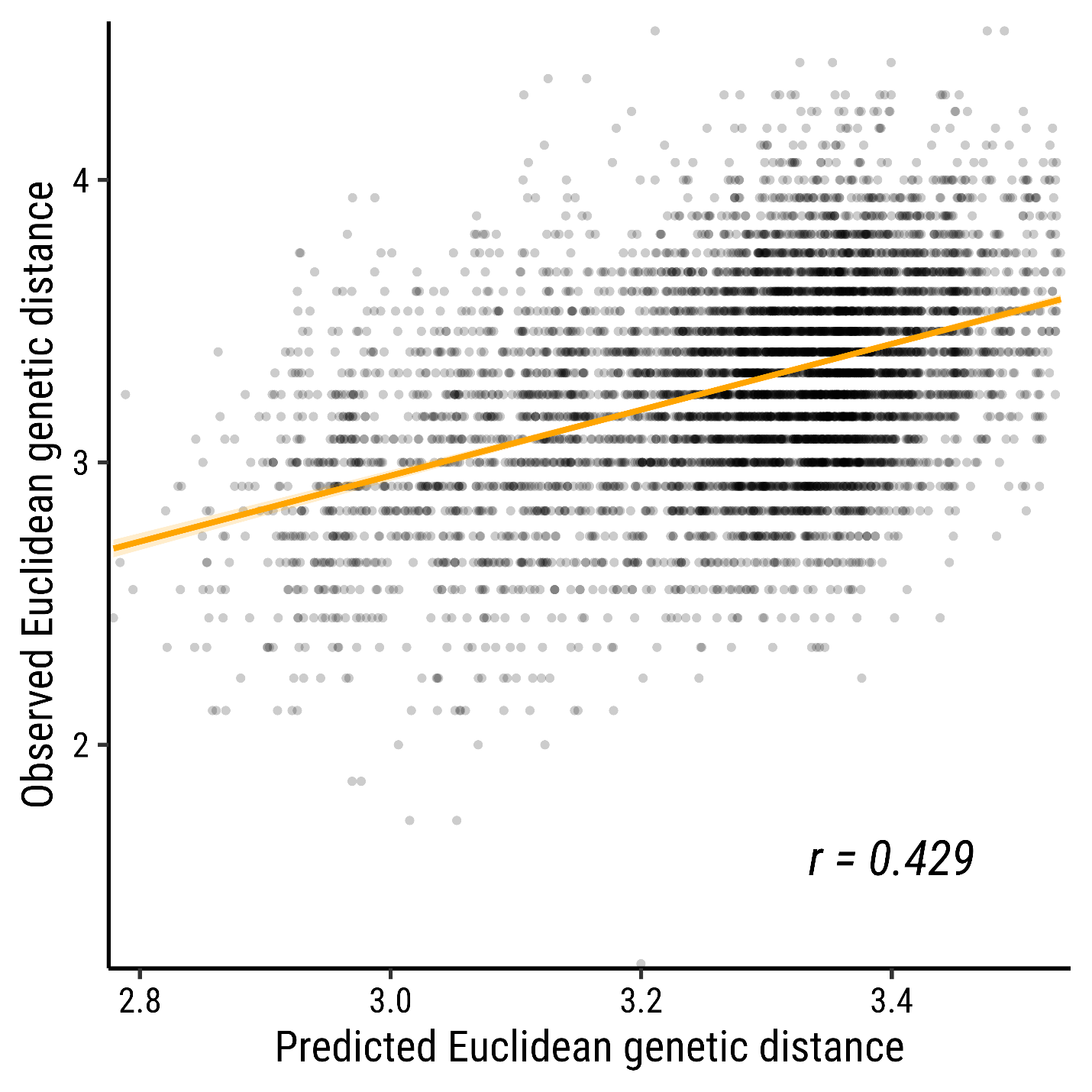


Figure S14. Scatterplot showing the relationship and Pearson’s correlation between observed Euclidean genetic distance and predicted Euclidean genetic distance by the top (straight-line) GBM model for 6,514 pairwise combinations of genetic samples from 116 American badgers (*Taxidea taxus jeffersonii*) collected from 2008 to 2023 in southern British Columbia, Canada.

**
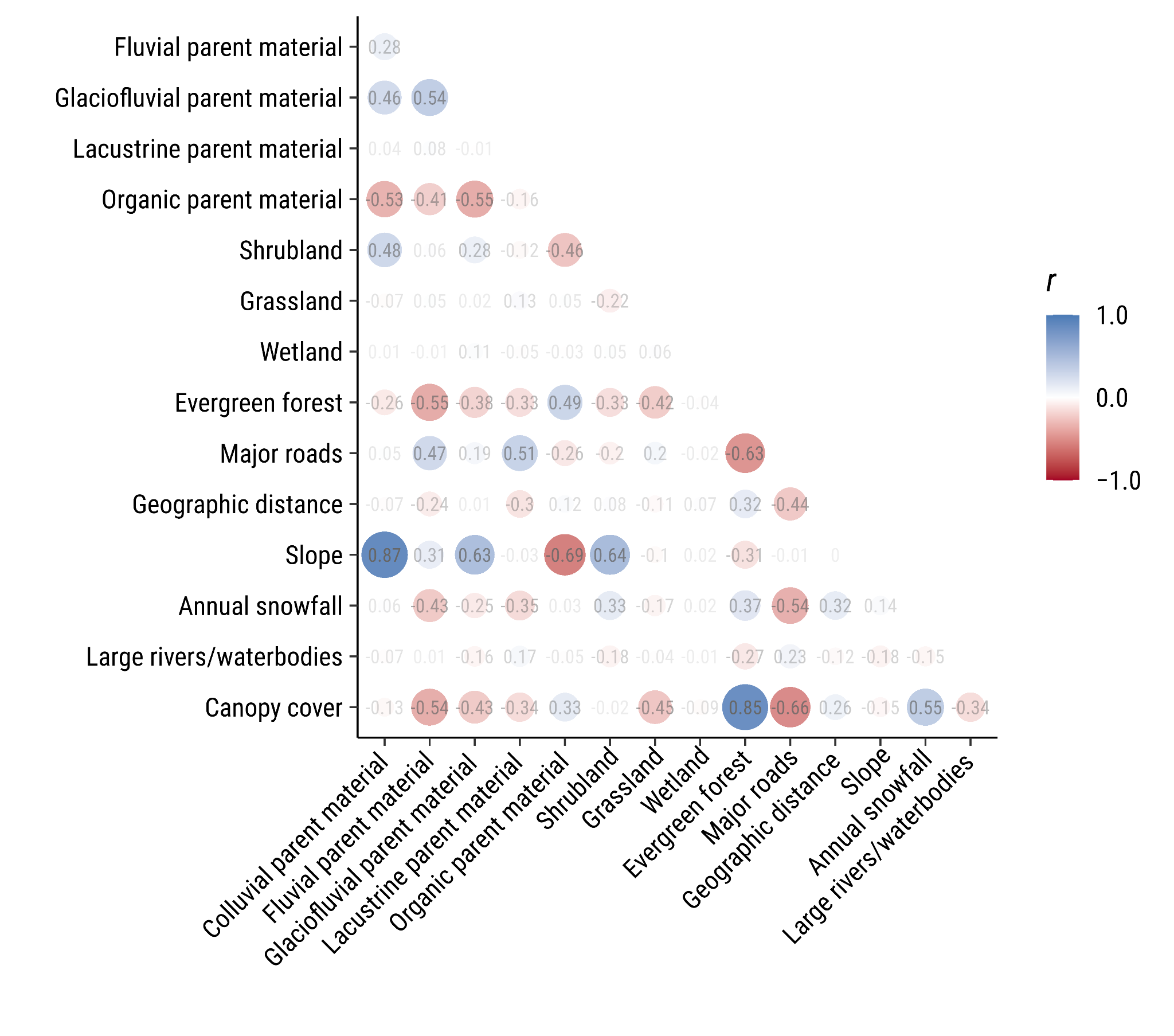
**

Figure S15. Multicollinearity (Pearson’s correlation) among explanatory variables (mean values) extracted from 500-m buffered straight lines between pairs of genetic sample locations from 116 American badgers (*Taxidea taxus jeffersonii*) from 2008 to 2023 in southern British Columbia, Canada. All parent material and land cover variables, along with major roads, were binary variables.**
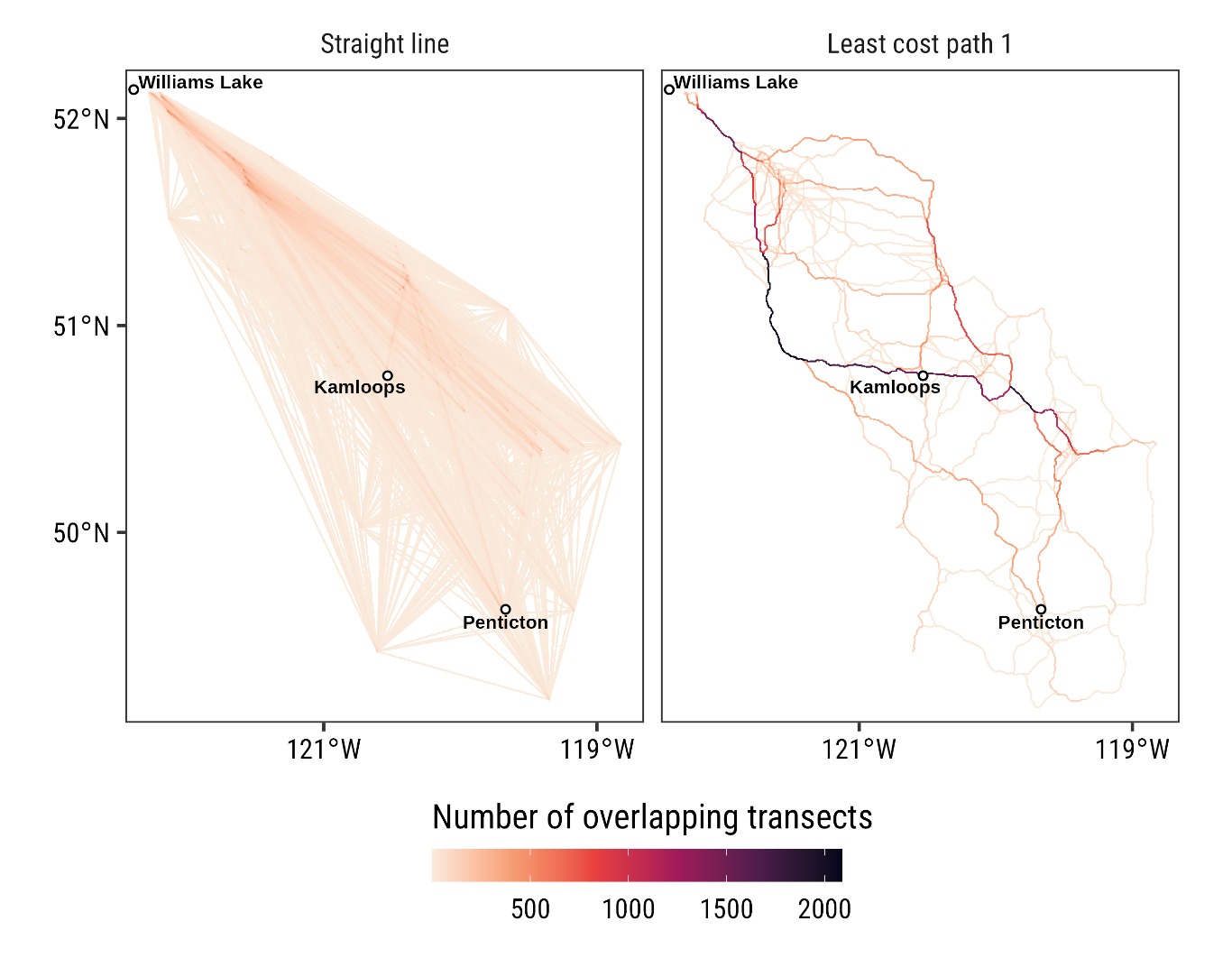
**

Figure S16. The number of overlapping 500-m wide transects connecting pairs of genetic sample locations from 116 American badgers (*Taxidea taxus jeffersonii*) from 2008 to 2023 in southern British Columbia, Canada. Mean values for all landscape covariates were extracted within these transects and used in gradient boosting machine models to predict pairwise genetic distance. The left panel depicts transects used for the straight-line (top performing) model, while the right panel depicts transects used for the first least cost path model.
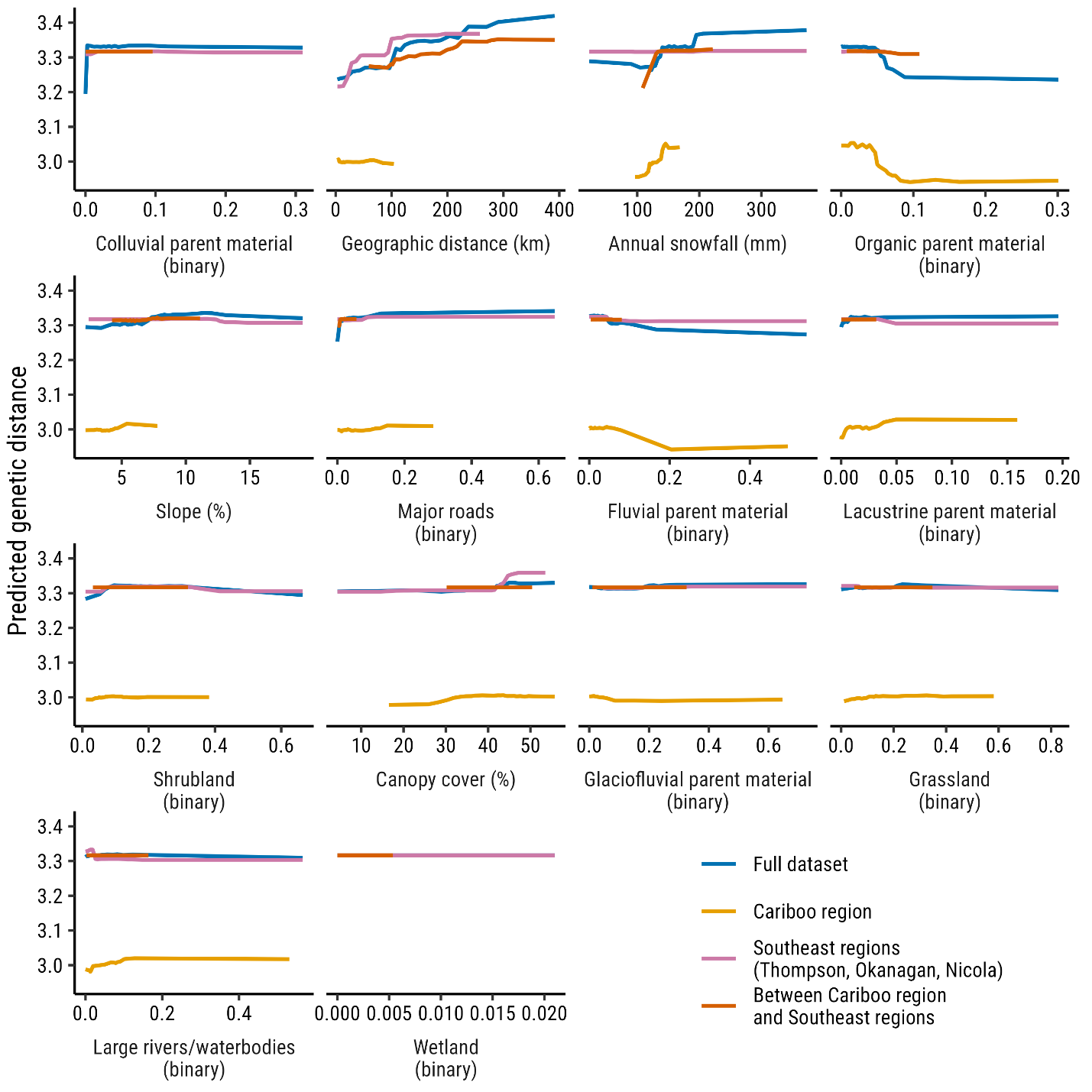


Figure S17. Accumulated local effects (ALE) plots showing the predicted Euclidean genetic distance across a range of values for all landscape variables (without variable selection) hypothesized to influence genetic connectivity from three gradient boosting models using data from 116 American badgers (*Taxidea taxus jeffersonii*) from 2008 to 2023 in southern British Columbia, Canada. Results are displayed separately for straight-line models fit using the full dataset, only those within the Cariboo (northwest) region, and only those within the Thomspon, Okanagan and Nicola (southeast) regions. Covariates for this model were average values extracted along 500-m buffered straight lines between pairs of genetic sample locations. Colluvial parent material and wetland did not exist along corridors in the Cariboo Region. Flat lines for most variables in the southeast regions indicate these variables had relatively little predictive power.
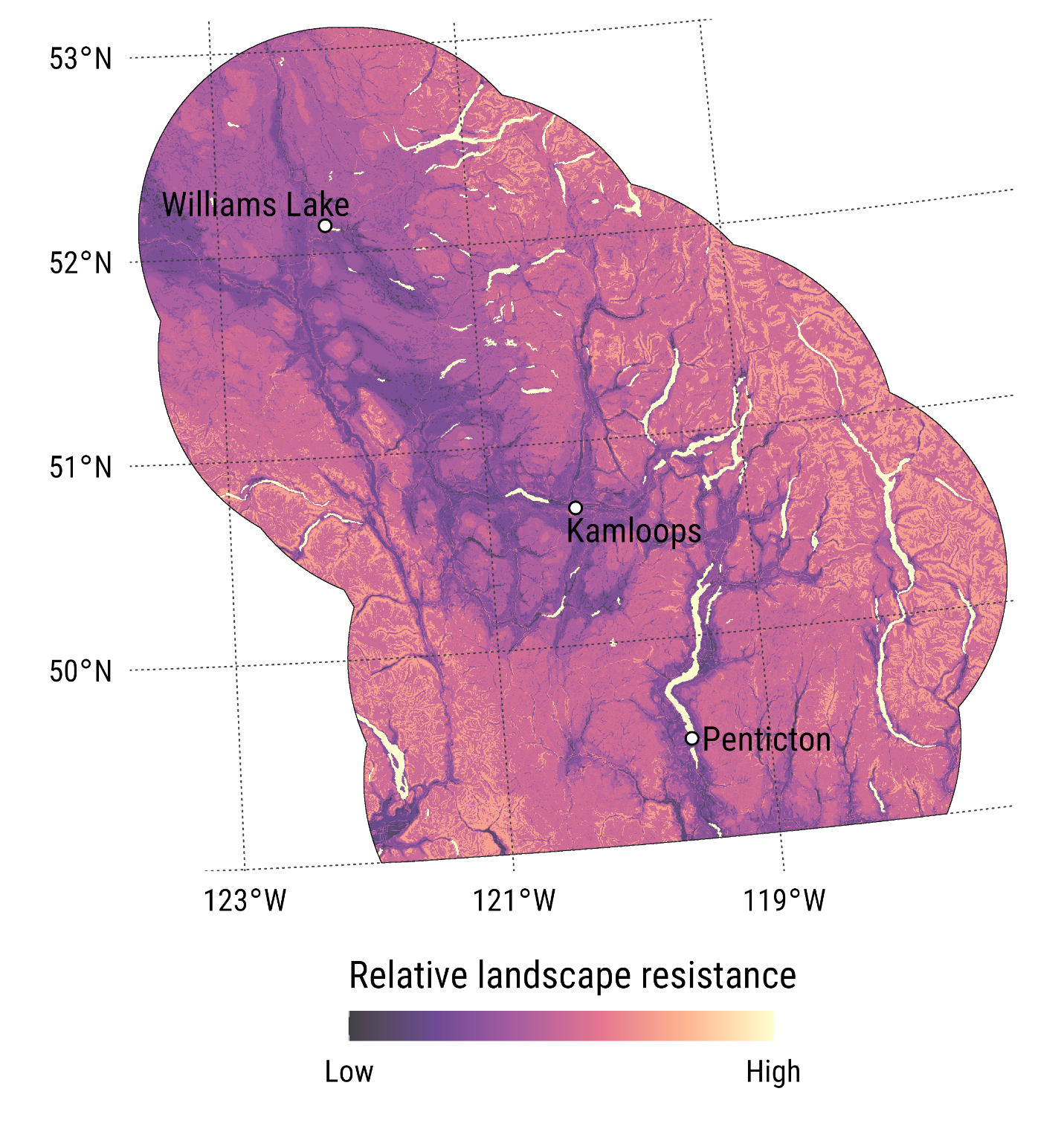
Figure S18. Predicted landscape resistance from the top gradient boosting machine model using data from 116 American badger (*Taxidea taxus jeffersonii*) genetic samples collected from 2008 to 2023 in southern British Columbia.


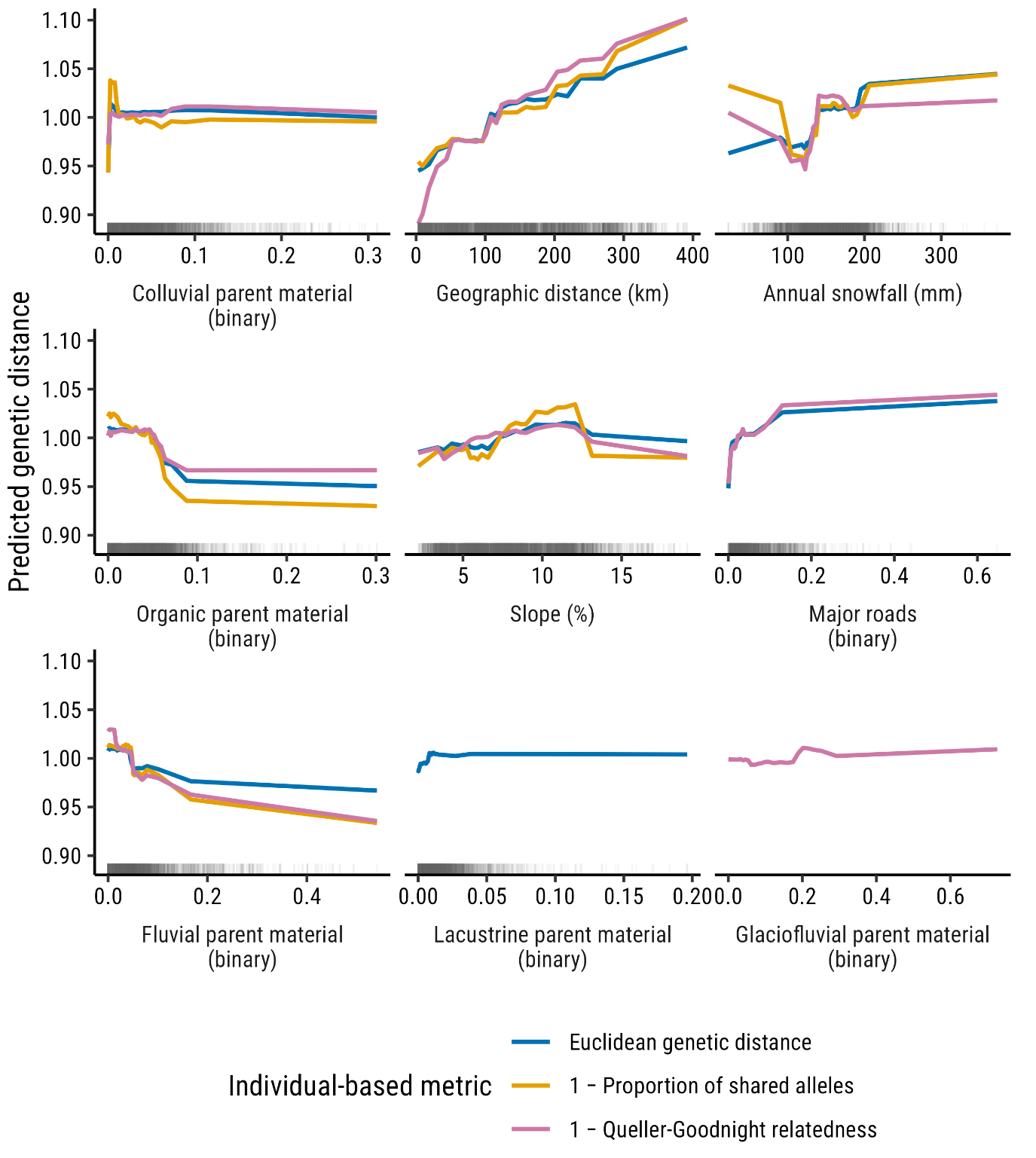


Figure S19. Accumulated local effects (ALE) plots comparing the predicted genetic distance (or relatedness) for three individual based metrics across a range of values for landscape variables hypothesized to influence genetic connectivity from gradient boosting models using data from 116 American badgers (*Taxidea taxus jeffersonii*) from 2008 to 2023 in southern British Columbia, Canada. To facilitate the visual comparison between metrics (which have different scales), each metric was normalized between 0 and 1 prior to modeling and predicted values (on the y-axis) were divided by their respective means for plotting. Variables in these models were average values extracted along 500-m buffered straight lines between pairs of genetic sample locations. Only variables that were retained in the variable selection process were included.
